# Enhancing autonomous agriculture control systems in greenhouses for sustainable resource usage using deep learning techniques

**DOI:** 10.1371/journal.pone.0344946

**Published:** 2026-03-26

**Authors:** Iman Hindi, Adham Alsharkawi, Malik Al-Ajlouni, Bassam Qarallah

**Affiliations:** 1 Industrial Engineering Department, Al Hussein Technical University, Amman, Jordan; 2 Computer Engineering Department, The University of Jordan, Amman, Jordan; 3 Mechatronics Engineering Department, The University of Jordan, Amman, Jordan; 4 Department of Horticulture and Crop Science, The University of Jordan, Amman, Jordan; Dr Shakuntala Misra National Rehabilitation University, INDIA

## Abstract

Greenhouse climate control is essential for optimizing crop growth while minimizing resource consumption in controlled environment agriculture. Traditional rule-based and fixed-action strategies often struggle to achieve a balance between these objectives. This paper proposes a reinforcement learning (RL) based framework for greenhouse climate control, integrating deep learning models to predict both crop growth and resource consumption. The framework enables an RL agent to optimize greenhouse control setpoints dynamically, maximizing crop yield while ensuring sustainable resource usage. The proposed system incorporates a Multi-Layer Perceptron (MLP) model to predict internal greenhouse climate conditions, a Long Short-Term Memory (LSTM) model for crop parameter estimation, and a separate LSTM model for forecasting daily resource consumption. These models collectively simulate a greenhouse environment where an RL agent learns to regulate temperature, CO_2_ concentration, and irrigation levels by interacting with the virtual environment. A custom reward function is designed to guide the agent, considering key crop parameters; stem elongation, stem thickness, and cumulative trusses; alongside resource consumption metrics, including heating, electricity, CO_2_, and irrigation costs. To enhance the adaptability of the RL agent, a feature-selection mechanism identified the most influential climate and control features, reducing observation complexity and accelerating convergence. Retraining under stochastic weather conditions strengthened robustness to dynamic environments, enabling the agent to consistently outperform fixed-action strategies. Evaluation revealed a stable Pareto frontier between yield and resource consumption, confirming that the framework accurately captured the productivity and sustainability trade-off and remained robust across varying reward-weight settings. Comparative analysis of multiple RL algorithms; Proximal Policy Optimization (PPO), Deep Deterministic Policy Gradient (DDPG), Soft Actor-Critic (SAC), and Twin Delayed Deep Deterministic Policy Gradient (TD3) demonstrated that TD3 outperforms other algorithms, achieving the highest cumulative rewards and reaching optimal policies faster. Experimental evaluations demonstrate that the proposed TD3 RL-based greenhouse control system achieves higher crop yield growth rates while optimizing resource usage, outperforming conventional greenhouse control strategies. This study presents a novel data-driven, adaptive greenhouse management approach, bridging the gap between crop growth modeling and autonomous climate control, contributing to sustainable and intelligent agricultural practices.

## 1. Introduction

In the past decade, climate change has significantly impacted global agriculture, creating challenges that demand innovative solutions to ensure food security. As governments and organizations prioritize sustainable energy usage to mitigate carbon footprints, the agricultural sector has increasingly turned to greenhouse farming to address extreme weather conditions such as heat waves, frost, and heavy rainfall. Greenhouses offer a controlled environment that protects crops and optimizes growing conditions, making them an essential tool in meeting the food demands of a global population expected to increase by 2.3 billion by 2050 [1].

Jordan, one of the world’s most water-scarce nations, faces unique agricultural challenges due to its arid climate, limited water resources, and high sensitivity to climate variability. Agriculture in Jordan consumes 51% of the country’s available water [2], posing critical concerns about resource sustainability. As climatic pressures increase, the need for innovative farming solutions and efficient resource management becomes more urgent. Greenhouse farming is particularly promising in Jordan as it enhances agricultural productivity while mitigating external climate-related constraints. However, the efficient management of greenhouse environments presents significant complexity, requiring real-time adjustments to parameters such as light, water, temperature, CO_2_ levels, and nutrients to meet plant growth needs.

Traditional manual control methods often fall short, leading to inefficient resource usage, suboptimal crop yields, and reliance on experienced growers, which are scarce in Jordan. To overcome these challenges, the agricultural sector is increasingly adopting advanced technologies, including artificial intelligence (AI), deep learning, and reinforcement learning (RL) [3]. These technologies enable farmers to improve decision-making, enhance resource efficiency, and minimize environmental impacts, while ensuring higher crop productivity [4].

This study presents a novel AI-based solution to automate and optimize greenhouse climate control. The proposed approach integrates reinforcement learning (RL) with ma- chine learning models to predict crop growth and resource consumption, providing a robust framework for real-time control of greenhouse parameters. By continuously monitoring and adjusting factors like temperature, irrigation, and CO_2_ levels, the RL agent optimizes plant growth and reduces unnecessary resource use. Additionally, a feature selection mechanism is incorporated to enhance model efficiency by focusing on the most influential predictors, thereby simplifying the training process while maintaining accuracy. Key contributions of this research include:

- Improved Productivity: Maximizes crop yields through optimal greenhouse control.
- Resource Efficiency: Reduces excessive use of critical resources like water, energy, and CO_2_ while lowering operational costs.
- Climate Resilience: Enhances the ability of greenhouses to withstand extreme weather conditions, ensuring stable crop production.
- Generalizability: Provides insights that are applicable to different climatic conditions, extending the utility of the proposed framework.
- Enhanced Research Capability: Offers tools for monitoring plant health and studying the impact of environmental variables on crop growth.

The primary objective of this study is to develop a robust, AI-driven greenhouse control system capable of improving crop yields and resource efficiency. Specific objectives include:

- Designing a Robust AI Model: Developing an AI model that can adapt to varying climatic conditions to optimize greenhouse control
- Addressing System Complexity: Using reinforcement learning algorithms to identify optimal control policies in non-linear, dynamic greenhouse environments.
- Resource and Crop Growth Prediction: Employing Long Short-Term Memory (LSTM) models for forecasting daily resource consumption and crop growth parameters.
- Integrating Feature Selection: Enhancing model efficiency by selecting the most influential features, thereby reducing computational complexity while preserving performance.

The research assumes the dataset used is recorded from advanced greenhouse infrastructure, including sensors and actuators for monitoring and controlling the environment, and uniform soil structure ensuring consistent water and nutrient distribution. The research focuses on cherry tomato cultivation as a representative crop for the study. Where the data sourced from the 2nd International Autonomous Greenhouse Challenge, providing a robust dataset for model training and evaluation [5] which contains the data from six teams participating in the Autonomous Greenhouse Challenge [6].

This research presents an innovative framework integrating reinforcement learning, deep learning, and feature selection to optimize greenhouse climate control, addressing critical challenges in controlled environment agriculture. By leveraging AI-driven approaches, the study tackles complexities such as resource overuse, climatic variability, and the scarcity of experienced growers, enabling the optimization of crop yields while ensuring sustainable resource efficiency. This work bridges the gap between advanced AI solutions and practical implementation, paving the way for more sustainable agricultural practices in greenhouse management.

## 2. Literature review

Increasing crop yield quantity and quality has been a primary focus for researchers globally. Efforts range from developing smart agricultural monitoring systems to designing robust frameworks that accurately detect plant needs based on critical factors such as soil moisture, weather conditions, and irrigation levels. The recent emergence of AI-based technologies, particularly machine learning (ML) and reinforcement learning (RL), has driven significant advancements in this domain. These technologies aim to replace traditional control systems, offering precision and efficiency in addressing the optimization challenges inherent in greenhouse environments.

### 2.1. Irrigation efficiency and smart watering

Efficient water management is critical for plant health. Researchers have employed IoT systems and AI algorithms to optimize irrigation processes. For instance, Gutiérrez-Gordillo et al. [7] identified leaf diameter as a key indicator of water stress. Data collection from IoT sensors has been used to train models, such as ensemble learning approaches [8–10] decision trees [11], and K-Nearest Neighbors (KNN) [12], for irrigation prediction. These models rely on soil moisture and weather data to determine water needs accurately.

In research of [13–15], the data collected used the moisture levels for irrigation control (On/Off) using predefined threshold. And they study various machine learning algorithms. Sema et.al, used IoT system to collect farming data and they used ANN (Artificial Neural Networks) as an irrigation system controller, which is trained as a classifier to turn the pump On/Off based on different parameter ranges of soil moisture, air humidity, and air temperature [16].

Ben Abdallah et al. [17] utilized Random Forest models combined with FAO’s CLIMWAT tool [18] to estimate water requirements with high accuracy (RMSE = 0.0509, R2 = 0.99). However, mathematical modeling limits real-world adaptability, as noted by researchers using deep learning classifiers for soil moisture conditions [19]. While in [20] paper, the authors used an estimation of evapotranspiration (ETMPT) from the infrared thermometry to assess the determination of variable rate irrigation (VRI).

LSTM-based systems have also emerged for predictive irrigation. Lyu et al. [21] and Kashyap et al. [22] demonstrated LSTM’s ability to forecast soil moisture and water needs. Reinforcement learning (RL) models, such as deep Q-networks, have further enhanced irrigation optimization. Alibabaei et al. [23] trained RL agents to maximize crop yield while minimizing water usage. Their comparison of on-policy and off-policy RL approaches revealed that on-policy methods reduced water consumption by 20% [24].

Kamyshova et al. [25] proposed an innovative Phyto-indicator-based approach using computer vision for irrigation prescriptions, while Amir et al. [26] and Yumang et al. [27] focused on greenhouse-specific systems for cherry tomato irrigation using machine learning and fuzzy logic, respectively. Hindi et al. [28] introduced a cascaded-output ANN model, achieving superior irrigation scheduling accuracy using the data of second International Greenhouse Challenge.

### 2.2. Monitoring plant status and growth rate

Monitoring plant health and growth stages is vital for decision-making. Advanced imaging and AI techniques have been widely adopted. Petropoulou et al. [29] and Lin et al. [30] utilized depth imaging and segmentation algorithms for lettuce growth monitoring, optimizing plant spacing and harvest timing. A two-stage CNN architecture by Gang et al. [31] estimated greenhouse lettuce growth indices such as fresh weight and leaf area with high accuracy.

Zhang et al. [32] developed a U-net mapping model for phenotypic parameter extraction from point clouds, demonstrating high performance in biomass estimation. These advancements facilitate precise growth predictions and enhance crop management.

Recent studies also highlight the role of Digital Twin frameworks integrated with RL to monitor crop growth and adapt control strategies in real-time [33].

### 2.3. Agricultural environmental modeling

Agricultural environmental modeling integrates crop simulation and machine learning to address changing environmental conditions. Traditional models like DSSAT and APSIM simulate crop growth based on environmental variables. Recent advancements incorporate machine learning for improved predictions [34]. For example, Van Klompenburg et al. highlighted the synergy between machine learning and simulation models [35].

Recent agricultural intelligence studies have continued to advance predictive and control methodologies beyond conventional statistical models. Sha et al. [36] proposed the ZHPO-LightXBoost model to handle limited-sample prediction problems for pesticide residues, demonstrating how hybrid machine-learning pipelines can maintain accuracy under data scarcity, an issue also critical to greenhouse control. Similarly, Gong et al. [37] introduced a LiDAR–Inertial–Ultrasonic SLAM system for plant-factory automation, exemplifying precise environmental sensing and autonomous navigation in controlled cultivation environments. These advances reflect the broader frontier of AI-driven agriculture, where data efficiency and automation are central to sustainability goals.

Moreover, IoT and cloud-based greenhouse monitoring systems are gaining traction [38], enabling real-time environmental monitoring and precision control while reducing human intervention.

Digital Twin technology, as reviewed by Purcell et al., creates virtual agricultural system replicas, optimizing real-time decisions [39]. Multi-objective optimization frameworks combine genetic algorithms with simulation models to balance environmental and economic goals [40].

Beyond agriculture, emerging AI frameworks such as the Knowledge-driven Two-stage Modulation Network (KTMN) by Shi et al. [41] illustrate progress in multi-modal learning and adaptive reasoning. Although developed for visual question answering, such adaptive knowledge-fusion techniques share conceptual similarities with reinforcement-learning frameworks that balance multiple dynamic objectives.

### 2.4. Autonomous farm control systems

The integration of AI and automation has transformed greenhouse management. RL- based frameworks are at the forefront of this innovation. Iacomi et al. [42] demonstrated a double deep Q-learning model for irrigation and chemigation, optimizing resource use and crop yield. Similarly, DRL models like DDPG have been applied for automatic sugar beet control [43]. While Elavarasan and Vincent developed a Deep Recurrent Q-Network model, which integrates recurrent neural networks with Q-learning to predict crop yields [44].

Recent works have expanded RL applications with model predictive control (MPC) to enhance robustness under uncertainty. Mallick et al. [45] and Morcego Seix et al. [46] proposed RL-MPC hybrid models for greenhouse climate control, outperforming traditional MPC under complex and stochastic environments.

Notable advancements include the CropGym environment for RL applications in nitrogen management [47] and DRLIC systems for irrigation scheduling using wireless sensor networks [48]. Kerkhof and Keviczky’s predictive control models for cherry tomato greenhouses combined data-driven approaches with meteorological data [49].

Additionally, Lee et al. presented an AI-powered greenhouse system (AI-GECS) that integrates weather forecasts and crop physiological indicators, employing a hybrid CLSTM-CNN-BP model to optimize microclimate control [50]. Astiaso Garcia showed that AI-driven greenhouse systems substantially improve energy efficiency, though challenges remain in CO_2_ and water usage optimization [51].

Furthermore, Fan et al. [52] investigated novel deep eutectic solvents with high CO₂ adsorption capacity, emphasizing the importance of efficient carbon-management solutions. Their findings conceptually align with this study’s emphasis on optimizing CO₂ utilization within greenhouse systems to achieve sustainable climate control.

Platero-Horcajadas et al. demonstrated the successful integration of IoT technologies with RL for greenhouse control, achieving energy savings and reducing the need for human intervention [53]. Such hybrid approaches combining RL, IoT, MPC, and Digital Twins are shaping the future of autonomous greenhouse management, driving toward more sustainable and resilient agriculture.

Recent studies on greenhouse automation have explored reinforcement learning for climate control, model predictive control based on simplified crop or energy models, and digital twin–driven optimization frameworks. While these approaches demonstrate promising improvements in isolated objectives, such as temperature regulation, energy efficiency, or yield maximization; they typically rely on single-objective formulations, static or manually tuned policies, or computationally intensive physics-based models.

In contrast, the present work advances the state of the art by integrating data-driven predictive models (MLP for climate dynamics and LSTM for crop growth and resource consumption) within a unified multi-objective reinforcement learning framework. This design enables explicit and interpretable trade-offs between productivity and sustainability, robustness to stochastic weather variability, and practical feasibility for deployment without reliance on high-fidelity digital twins.

Our RL-based greenhouse framework advances prior work by enabling adaptive, data-driven control in uncertain and dynamic environments. LSTM models forecast climate conditions, crop growth, and resource consumption, providing accurate predictive signals for decision making.Reinforcement learning agents then optimize control policies that balance yield with resource efficiency, adapting dynamically to stochastic weather and operational variability. This integrated approach enhances robustness, captures the yield-resource trade-off, and addresses key sustainability challenges in modern greenhouse management.

## 3. Dataset description

The dataset utilized in this research originates from the 2nd International Autonomous Greenhouse Challenge (2022), conducted at the Greenhouse Horticulture Business Unit, Wageningen Research, in Bleiswijk, The Netherlands. This dataset provides comprehensive time-series data collected over a six-month period of cherry tomato production across six high-tech greenhouse compartments. It includes external meteorological data, greenhouse climate conditions, climate control setpoints, actuator responses, daily resource consumption, and crop growth observations.

The dataset was generated during a competition where five international teams; The Automators, AICU, IUA.CAAS, Digilog, and Automatoes; developed AI-driven strategies to autonomously manage greenhouse operations. Their objective was to maximize net profit by minimizing resource consumption (water, energy, CO_2_) while ensuring optimal crop yield and quality. The AI-controlled compartments were compared against a manually operated reference compartment, managed by experienced Dutch growers, providing a benchmark for evaluating AI-driven greenhouse management.

The dataset includes both raw and processed sensor data, capturing climate control actions, irrigation metrics, actuator statuses, and realized setpoints. Advanced sensor networks and climate measurement devices were employed, complemented by manual crop growth observations and irrigation sample analyses. This dataset serves as a valuable foundation for training and validating AI models, supporting the development of reinforcement learning- based greenhouse control frameworks for sustainable and optimized agricultural practices.

## 4. Methodology

**Fig 1:**
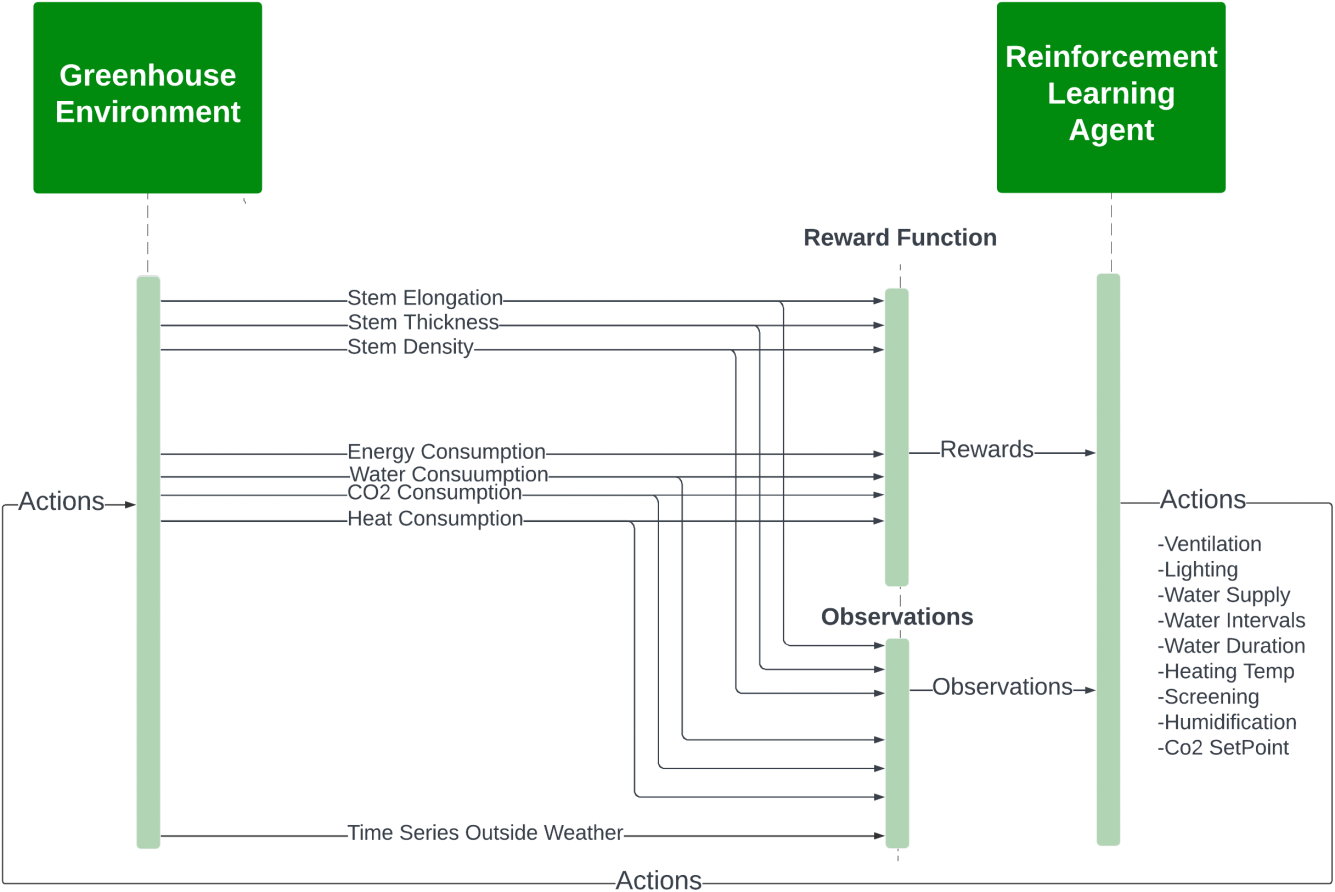
The structure of the proposed methodology for autonomous greenhouse control system.

The methodology employed in this study is illustrated in Fig 1 outlining the structure of the proposed autonomous greenhouse control system. The approach is organized into two main components: The first is a virtual environment for greenhouse climate, crop parameters, and resource consumption estimations, and the second is a reinforcement learning (RL) agent for optimizing greenhouse setpoints.

The autonomous control cycle begins with sensor acquisition of external weather and internal climate; the data enter the MLP-based climate estimation and LSTM-based crop/ resource forecasting. The estimated observations enter the RL agent that determines weekly setpoints for CO₂, heating, ventilation, and irrigation, completing a closed-loop optimization of yield and sustainability.

### 4.1. Virtual environment simulator

**Fig 2:**
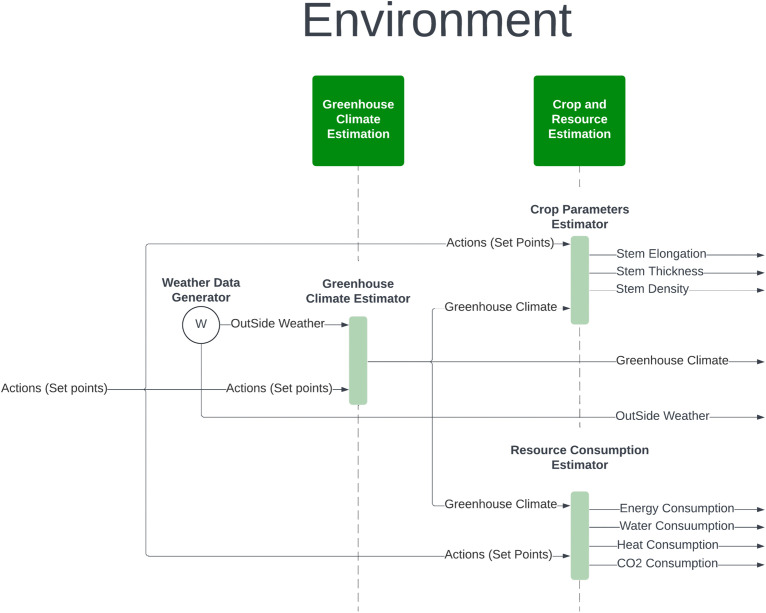
The structure of the proposed virtual environment simulator for autonomous greenhouse control system.

The virtual environment simulates greenhouse operations by integrating machine learning models to estimate outcomes based on external weather and control setpoints. Fig 2 presents the virtual environment architecture, consisting of:

- Greenhouse Climate Estimation: An MLP model predicts greenhouse climate based on outside weather and control setpoints.
- Crop Parameter Estimation: An LSTM model predicts weekly crop parameters, including stem elongation, thickness, and cumulative trusses.
- Resource Consumption Estimation: Another LSTM model predicts daily resource consumption, such as heating, CO_2_ usage, electricity, and irrigation water.

The environment enables RL agents to train by simulating realistic greenhouse dynamics, facilitating the optimization of crop yield and resource efficiency.

#### 4.1.1. Greenhouse climate estimation.

**Fig 3:**
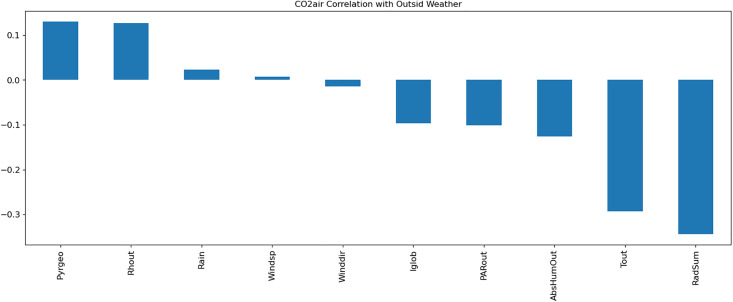
Visualization of the correlation of CO_2_air with outside weather parameters.

**Fig 4:**
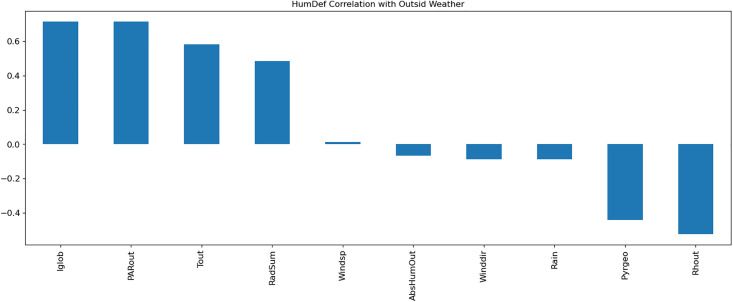
Visualization of the correlation of HumDef with outside weather parameters.

**Fig 5:**
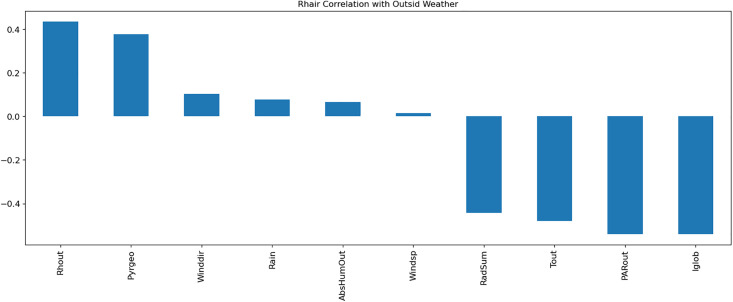
Visualization of the correlation of Rhair with outside weather parameters.

**Fig 6:**
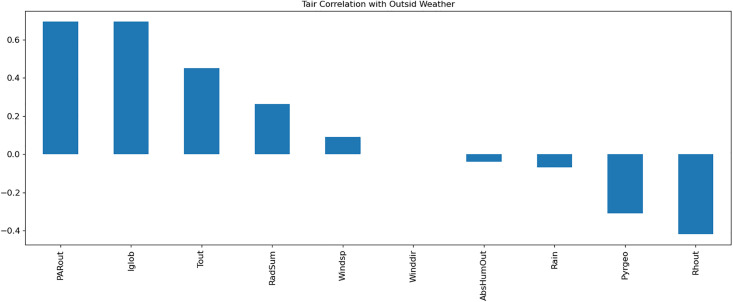
Visualization of the correlation of Tair with outside weather parameters.

**Fig 7:**
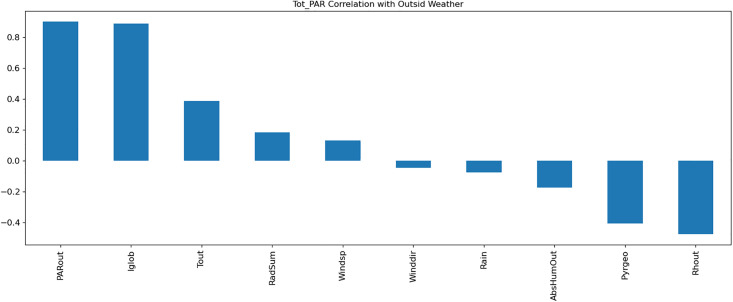
Visualization of the correlation of Total_PAR with outside weather parameters.

A Multi-Layer Perceptron (MLP) model was developed to predict greenhouse climate parameters. Tables 1–3 detail the external weather and control setpoints used as inputs, and greenhouse climate parameters as outputs. Data normalization, exploratory data analysis (EDA), and temporal correlation analyses (Figs 3–7) informed feature selection and model design.

**Table 1:** Outside weather parameters.

Parameter	Description	Unit
**Tout**	Outside temperature	°C
**Rhout**	Outside relative humidity	%
**Iglob**	Solar radiation	W/m²
**Windsp**	Wind speed	m/s
**Winddir**	Wind direction	[0–128]
**RadSum**	Radiation sum	J/cm²
**PARout**	PAR weather measurement	µmol/m²·s
**Pyrogeo**	Heat emission (pyrgeometer)	W/m²
**Rain**	Rain (status)	[1 = rain, 0 = dry]
**AbsHumOut**	Absolute humidity (outside)	g/m³

**Table 2:** Greenhouse control setpoints.

Control Setpoint	Description	Unit
**CO**_**2**_ **_sp**	CO₂ setpoint	ppm
**dx_sp**	Humidity deficit setpoint	g/m³
**t_rail_min_sp**	Rail pipe minimum temperature	°C
**t_grow_min_sp**	Crop pipe minimum temperature	°C
**Assim_sp**	Assimilation lighting (HPS)	% [0–100]
**scr_enrg_sp**	Energy curtain	% [0–100]
**scr_blck_sp**	Blackout curtain	% [0–100]
**t_heat_sp**	Heating temperature	°C
**t_vent_sp**	Ventilation temperature	°C
**window_pos_lee_sp**	Lee side window position	%
**water_sup_int_sp_min**	Water supply interval time	minutes
**int_blue_sp**	Intensity of blue spectrum	[0–1000]
**int_red_sp**	Intensity of red spectrum	[0–1000]
**int_farred_sp**	Intensity of far-red spectrum	[0–1000]
**int_white_sp**	Intensity of white spectrum	[0–1000]

**Table 3:** Greenhouse climate parameters.

Parameter	Description	Unit
**Tair**	Greenhouse air temperature	°C
**Rhair**	Greenhouse relative humidity	%
**CO**_**2**_ **air**	CO₂ concentration inside greenhouse	ppm
**HumDef**	Humidity deficit	g/m³
**VentLee**	Leeward vents opening	% [0–100]
**Ventwind**	Windward vents opening	% [0–100]
**AssimLight**	HPS lamps status (on/off)	% [0–100]
**EnScr**	Energy curtain opening	% [0–100]
**BlackScr**	Blackout curtain opening	% [0–100]
**PipeLow**	Rail pipe temperature	°C
**PipeGrow**	Crop pipe temperature	°C
**CO**_**2**_ **_dos**	CO₂ dosing	kg/ha·h
**Tot_PAR**	Total inside PAR	µmol/m²·s
**Tot_PAR_Lamps**	PAR sum from HPS and LED	µmol/m²·s
**EC_drain_PC**	Drain electrical conductivity (EC)	dS/m
**pH_drain_PC**	Drain pH	[-]
**Water_sup**	Cumulative minutes of irrigation	minutes
**Cum_irr**	Cumulative liters of irrigation	L/m²

**Fig 8:**
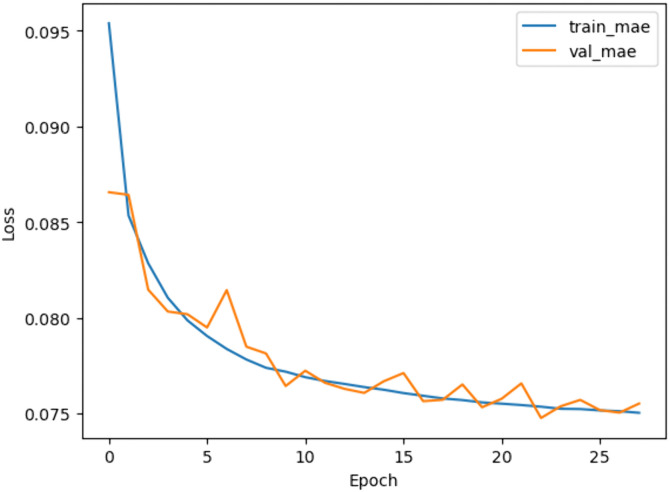
Training and validation loss curves over the epochs.

The MLP architecture consisted of two hidden layers with ReLU activation. The model achieved superior predictive accuracy (MAE = 0.06) on test data, as shown in the training curves in Fig 8.

#### 4.1.2. Crop parameter and resource consumption estimation.

**Fig 9:**
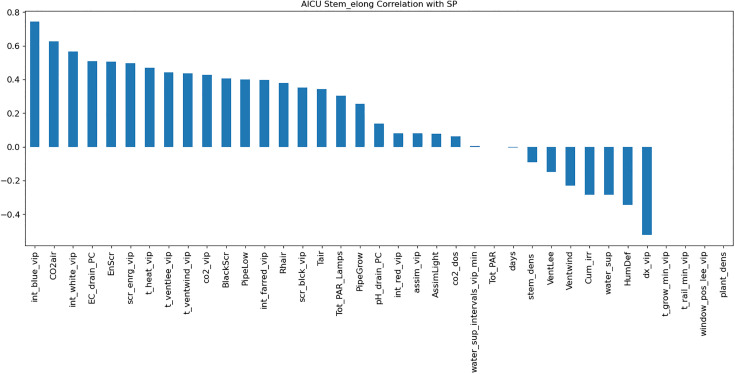
Correlation of Stem Elongation with greenhouse control setpoints.

An LSTM-based model was trained to predict weekly crop parameters Table 4 using time-series data after Normalization in range (0–1) using MinMaxScaler. The model architecture incorporated 1D convolutional layers for feature extraction and LSTM layers for sequential pattern learning. Feature importance analysis (Fig 9) using random forest guided the selection of key features, improving model performance. The LSTM model achieved an MAE of 0.16 on the test set (Table 5), outperforming the MLP model.

**Table 4:** Description of crop parameters and corresponding units.

Parameter	Description	Unit
**Stem_elong**	Stem growth per week, based on the average of 10 sampled stems	cm/week
**Stem_thick**	Stem thickness per week, based on the average of 10 sampled stems	mm/week
**Cum_truss**	Cumulative number of newly set trusses per stem (a truss is set when ≥5 flowers are set)	Weekly number/stem

**Table 5:** MAE test loss of crop parameters for trained LSTM and MLP models with and without feature selection.

Model	MAE loss (without feature selection)	MAE loss (with feature selection)
**MLP**	0.25	0.17
**LSTM**	0.20	0.16

**Fig 10:**
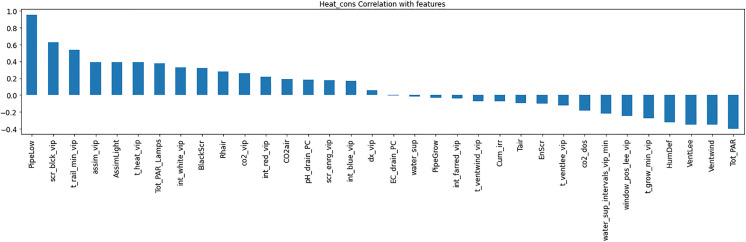
Correlation of Heat Consumption with climate setpoints.

**Fig 11:**
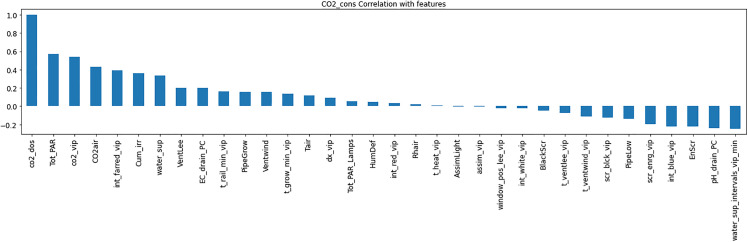
Correlation analysis for High Electrical Consumption.

**Fig 12:**
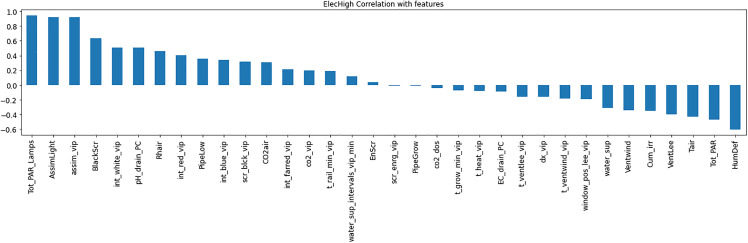
Low Electrical Consumption correlation with setpoints.

**Fig 13:**
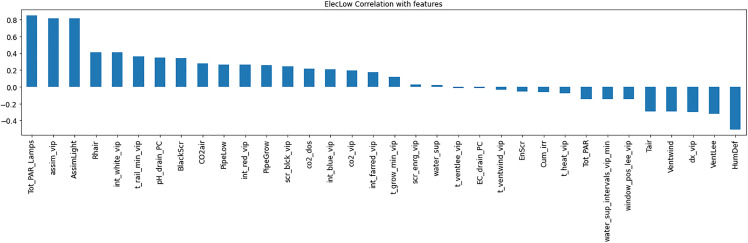
Correlation between CO_2_ Consumption with setpoints.

**Fig 14:**
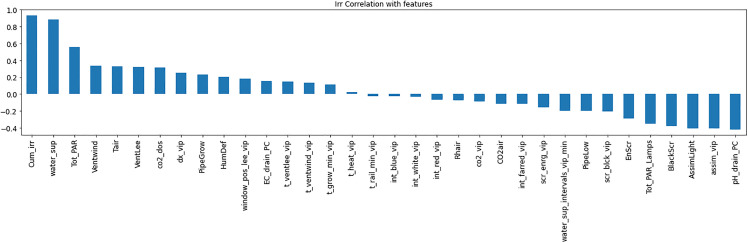
Irrigation Consumption correlation with setpoints.

**Fig 15:**
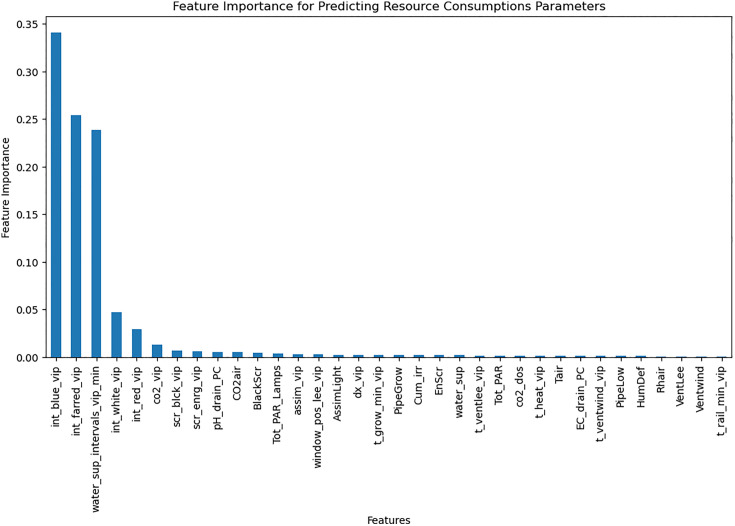
Important features for predicting resource consumption using random forest.

Resource consumption models predicted daily usage metrics, including heating, electricity, CO_2_, and irrigation (Table 6). The analysis of the feature importance (Figs 10–15) identified critical factors such as humidity, temperature, and irrigation intervals. The LSTM model demonstrated better performance (MAE = 0.16) compared to the MLP model (Table 7).

**Table 6:** Description of resource consumption parameters and corresponding units.

Parameter	Description	Unit
**Heat_cons**	Heating energy consumption	MJ/m²·day
**ElecHigh**	Electricity consumption during peak hours (07:00–23:00)	kWh/m²·day
**ElecLow**	Electricity consumption during off-peak hours	kWh/m²·day
**CO₂ consumption**	Computed based on CO₂ dosing	kg/m²·day
**Irr**	Cumulative daily irrigation	L/m²·day

**Table 7:** MAE loss of resource consumptions for trained LSTM and MLP models with and without feature selection on test set.

Model	MAE loss (without feature selection)	MAE loss (with feature selection)
**MLP**	0.17	0.17
**LSTM**	0.16	0.16

While the LSTM-based models in this study demonstrated strong predictive performance for both crop parameters and resource consumption, it is important to acknowledge that more advanced time series architectures may further enhance adaptability under complex and irregular environmental conditions. Recent studies, such as [54], have introduced Transformer-based models for dynamic system degradation prediction, highlighting their superiority over traditional RNNs in capturing long-term dependencies and irregular fluctuations. In future work, the LSTM framework presented here can be extended to include Transformer architecture, allowing a direct comparison of their adaptability to extreme weather sequences and environmental variability. Such exploration would provide additional robustness and improve resilience of greenhouse control systems under challenging scenarios.

#### 4.1.3. Reinforcement learning gym environment.

The designed reinforcement learning aimed to optimize greenhouse setpoints for temperature, humidity, CO_2_ levels, and irrigation, balancing crop yield and resource efficiency.

The interaction between models and the RL agent was designed around a weekly control cycle, consistent with crop growth and resource reporting in the dataset. Each environment step spans 2016 timesteps, corresponding to 5-minute intervals across 7 days. At the beginning of each step, the RL agent proposes a set of weekly control setpoints. These actions are combined with weather inputs and processed by the MLP climate estimator to predict greenhouse conditions at hourly resolution. The predicted climate variables and control setpoints are then used as inputs to two LSTM models: one forecasting weekly crop parameters and the other daily resource consumption, which is aggregated into weekly totals. The outputs of these models are synchronized by indexing the corresponding time window (2016 timesteps per week), ensuring that all predictions reflect the same control actions and environmental conditions. The updated state — including weather, climate, crop, and resource values is then provided back to the RL agent before the next decision step. This structure maintains temporal consistency between predictions and decisions while aligning the simulation with practical weekly greenhouse management cycles.

*Observation and Action Spaces:* The RL agent observed greenhouse climate, crop parameters, resource consumption, and external weather by state function as dictionary. The agent adjusted continuous setpoints (e.g., CO_2_, irrigation intervals, heating levels) to optimize greenhouse conditions (Table 8). These action setpoints are selected by the Agent from continuous range between (0–1).

**Table 8:** Action set points which the RL agent can take to control greenhouse environment.

Control setpoint	Description	Unit
CO_2_**_sp**	CO₂ setpoint	ppm
**scr_enrg_sp**	Energy curtain setpoint	% [0 or 100]
**scr_blck_sp**	Blackout curtain setpoint	% [0 or 100]
**t_heat_sp**	Heating temperature setpoint	°C
**t_vent_sp**	Ventilation temperature setpoint (leeward vents)	°C
**water_sup_int_sp_min**	Water supply interval time	minutes
**pH_drain_sp**	Drain pH	[-]
**int_white_sp**	Intensity of white spectrum channel	[0–1000]

*Reward Function:* Designing an effective reward function is pivotal for guiding a reinforcement learning (RL) agent toward achieving the dual objectives of maximizing crop growth and minimizing resource consumption in a greenhouse environment. The reward function must account for multiple objectives, balancing productivity and sustainability, while ensuring stable and effective control strategies.

The reward function (Equation 1) balanced crop growth metrics (stem elongation, thickness, trusses) with resource penalties (e.g., heating and irrigation). Efficiency factors and stability penalties ensured optimal and consistent control actions. The inclusion of incentives (e.g., additional rewards for high crop quality) further guided the agent’s learning.


$$reward=\alpha \times crop_{reward}-\beta \times resource_{penalty}+punishment+big_{reward}+\delta \times efficiency\;factor+\gamma stability\;penalty$$ (1)

Where: *α* = 1.0 and *β* = 0.2 are scaling factors for the crop reward and resource penalty, respectively, and *δ* = 0.01, *γ* = 0.001 are scaling factors for efficiency factor and stability penalty, respectively. Punishment and big rewards capture additional incentives or penalties based on crop and resource conditions.

The crop growth reward encourages the RL agent to enhance critical crop parameters, including Stem Elongation, Stem Thickness, and Cumulative Trusses.

Each parameter is normalized against its maximum value to ensure consistency across different scales. The crop growth reward, crop_reward, is computed as a weighted sum of these normalized values as equation 2:


$$crop_{reward}=w1\times \frac{Stem\;Elongation}{max\_stem\_elong}+w2\times \frac{Stem\;Thickness}{max\_stem\_thick}+w3\times \frac{Cumulative\;Trusses}{max\_cum\_trusses}\;$$ (2)

Where: *w*1 = 0.4, *w*2 = 0.3, and *w*3 = 0.3 are weights assigned to each parameter.

To discourage excessive use of resources, a resource penalty, the resource_penalty, is applied. The penalty considers key resources such as: Heat Consumption, CO_2_ Consumption, Electricity Consumption (High and Low), and Irrigation.

The penalty is computed as Equation 3 below:


$$resource\_penalty=p1\times \frac{Heat\;Consumption}{max\_heat}+p2\times \frac{CO^{2}\;Consumption}{max\_CO2}+p3\times \frac{Electricity\;Consumption}{max\_electricity}+p4\times \frac{Irrigation}{max\_irrigation}\;$$ (3)

Where: *p*1 = 0.2, *p*2 = 0.3, *p*3 = 0.2, and *p*4 = 0.3 are the respective weights that adjust the importance of each resource.

To guide the agent toward desirable outcomes, additional incentives and penalties are incorporated:

- Incentives for High Crop Quality: Rewards (+0.2) are given when individual crop parameters exceed thresholds (e.g., ≥ 0.7), with higher rewards (+1.0) for all parameters reaching (*≥*0.8).
- Penalties for Low Crop Quality: Penalties (*−*0.1) are imposed if any parameter falls below 0.5, with severe penalty (*−*1.0) for all parameters below 0.5.
- Resource Overuse Punishment: Extra penalties (1.0) are applied when resource consumption exceeds predefined thresholds, triggering an episode termination.

A stability penalty promotes smooth control actions by discouraging abrupt changes. It is calculated as the following equation 4:


$$stability_{penalty}=s\cdot \sum |action\left(t+1\right)-action\left(t\right)|$$ (4)

Where: S=0.001 is scaling factor for stability penalty.

The efficiency factor measures the agent’s ability to achieve crop growth with minimal resource use. It is computed as Equation 5:


$$efficiency_{factor}=\frac{crop\_reward}{1+resource\_penalty}\;$$ (5)

The weighting coefficients (α, β, w₁–₃, p₁–₄) were selected according to agronomic significance and pilot tuning. Greater weight was assigned to stem elongation and irrigation cost, which most directly influence biomass gain and resource sustainability.

**Fig 16:**
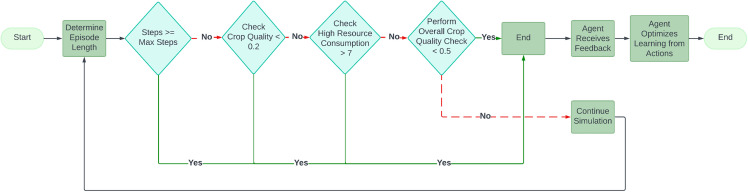
The structure of termination criteria during each episode.

*Termination criteria:* Termination criteria are employed to ensure sustainable greenhouse management by limiting episodes to 23 steps representing 23 weeks, enforcing crop quality thresholds, and penalizing excessive resource consumption. These criteria, illustrated in Fig 16, guide the agent’s learning by balancing crop productivity and resource efficiency.

#### 4.1.4. Environment enhancement with feature selection.

**Fig 17:**
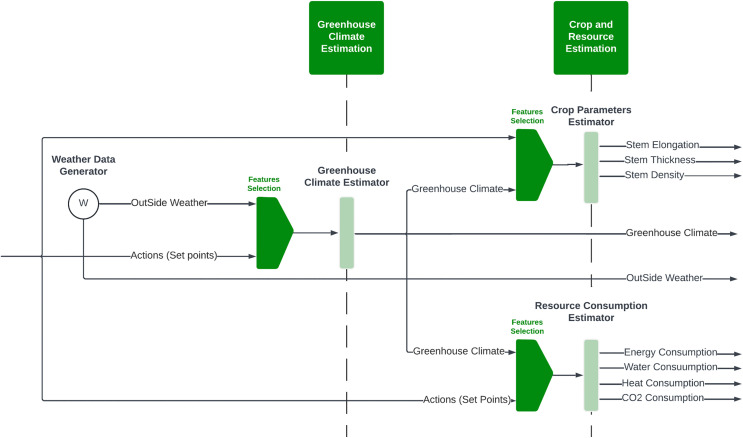
Enhanced RL environment structure with feature selection.

The enhancement process starts with evaluating the initial environment, which uses pre-trained estimators on all features, establishing a baseline for the RL agent’s training performance. A feature selection mechanism, using a random forest model, is then introduced to identify and focus on critical features, reducing system complexity. This maintains the agent’s baseline performance while improving learning efficiency by concentrating on significant features. Fig 17 illustrates the enhanced environment with feature selection integrated before each estimation process.

Feature importance was computed using Random Forest models, ensuring temporal stability of selected features across the control horizon. While alternative feature-selection approaches such as PCA or SHAP could be considered, Random Forest importance was selected for its robustness and interpretability in nonlinear, multivariate greenhouse environments.

**Fig 18:**
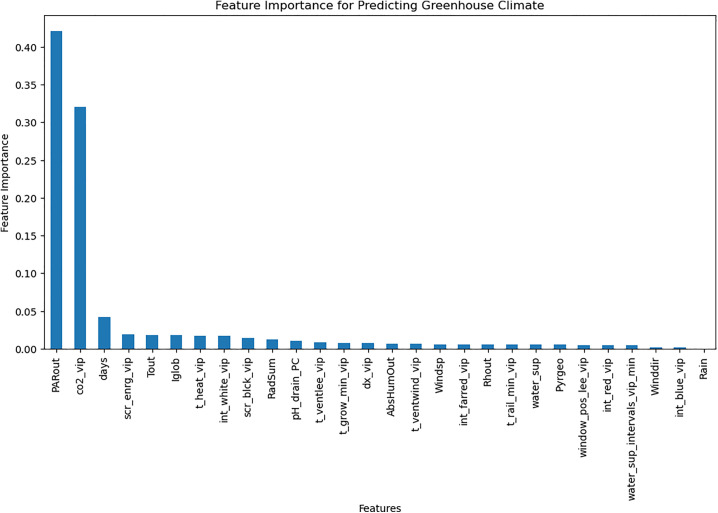
Feature importance for predicting greenhouse control parameters.

**Fig 19:**
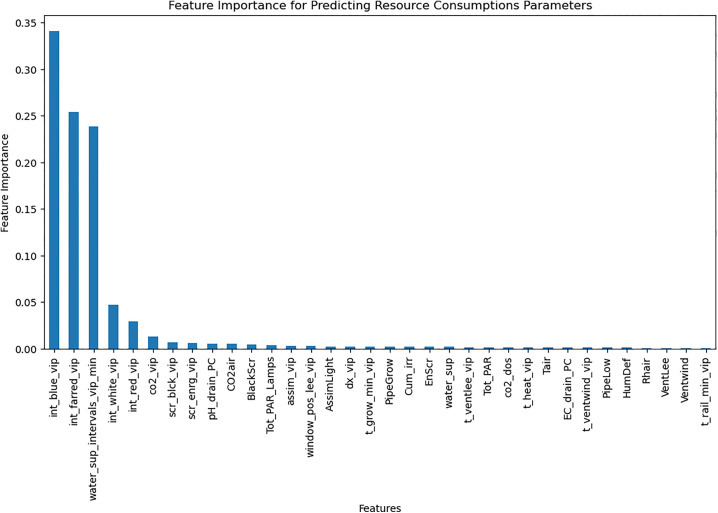
Feature importance distribution for resource consumption.

**Fig 20:**
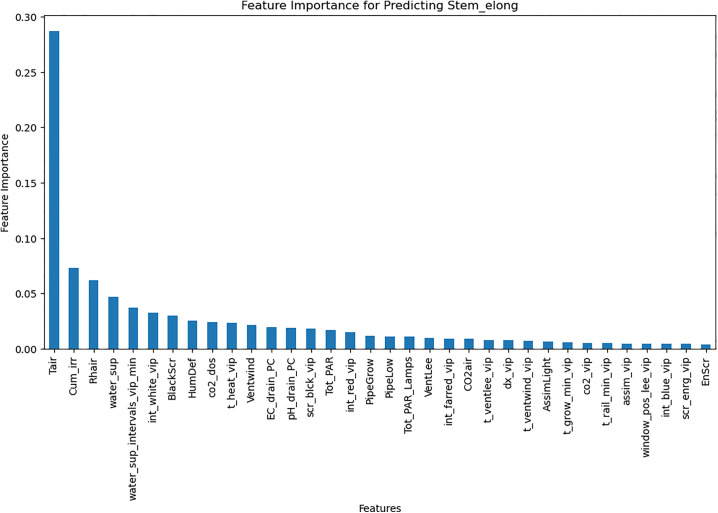
Feature importance for predicting all crop parameters.

**Fig 21:**
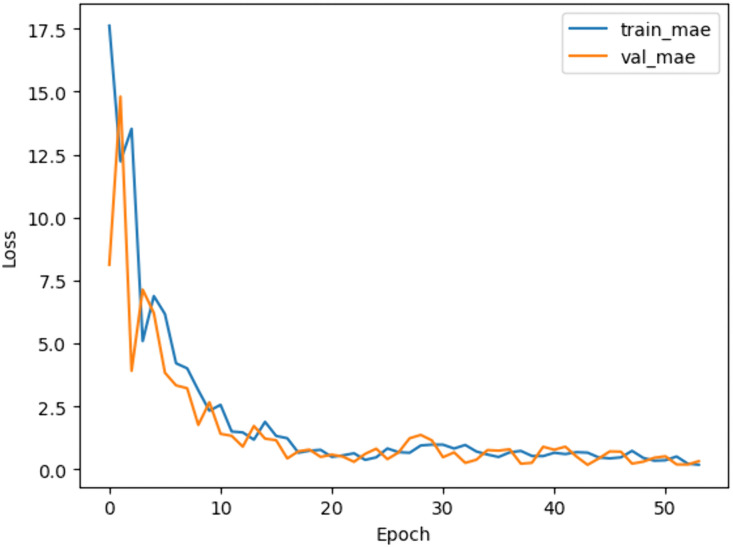
MLP model’s train and validation MAE loss overtraining epochs.

**Fig 22:**
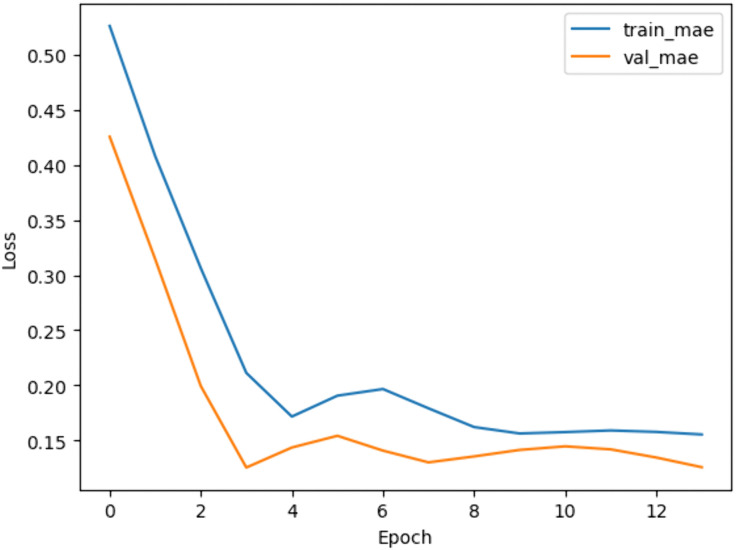
LSTM model train and validation MAE loss overtraining epochs.

The RL agent was then retrained on the environment after applying feature selection. Feature selection improved training efficiency by reducing models’ complexity. Figs 18–20 show the identified key features for each estimator using Random Forest importance analysis. The implemented feature selection mechanism using Random Forest importance ranking was used for each estimator (MLP climate prediction, LSTM crop growth, LSTM resource consumption). Models trained on selected features demonstrated faster convergence and lower loss values (Figs 21, 22).

The implemented feature selection mechanism using Random Forest importance ranking was used for each estimator (MLP climate prediction, LSTM crop growth, LSTM resource consumption), the top features were identified and used as inputs to reduce model complexity. Table 9 lists the top selected features per model, ranked by their importance scores. This selection preserved predictive performance while reducing observation space dimensionality, thereby accelerating RL agent convergence.

**Table 9:** Top selected features per model based on Random Forest importance ranking.

Estimator	Selected features
**Greenhouse control Estimator**	PARout, Tout, Iglob, RadSum, scr_enrg_vip, t_heat_vip, int_white_vip, scr_blck_vip, pH_drain_PC, CO_2__vip, t_ventlee_vip, days
**Resource consumption estimator**	Cum_irr, BlackScr, water_sup_intervals_vip_min, EC_drain_PC, pH_drain_PC, CO_2_air, dx_vip, HumDef, Rhair, days, Tot_PAR
**Crop parameters estimator**	Tair, pH_drain_PC, Cum_irr, t_heat_vip, dx_vip, Tot_PAR, water_sup_intervals_vip_min, PipeGrow, EC_drain_PC, BlackScr, CO2_dos, Tot_PAR_Lamps, scr_enrg_vip, Rhair, HumDef, days

#### 4.1.5. Environment enhancement with stochastic external weather.

The RL agent’s robustness was evaluated by extending the virtual environment to include random weather data generation. Initially tested with historical weather data, the environment was later modified to simulate dynamic and unpredictable conditions to assess the models’ adaptability. Observing a performance decline, the RL agent was re-trained in the enhanced environment, enabling it to learn strategies for adapting to stochastic weather patterns.

### 4.1. Training and evaluation of reinforcement learning algorithms

To address the challenges of greenhouse climate control, multiple reinforcement learning (RL) algorithms were implemented, trained, and compared within a virtual environment. The algorithms evaluated include Proximal Policy Optimization (PPO) [55], Deep Deterministic Policy Gradient (DDPG) [56], Soft Actor-Critic (SAC) [57], and Twin Delayed Deep Deterministic Policy Gradient (TD3) [58]. Among these, PPO was selected as the baseline reference model due to its stability and effectiveness in handling continuous action spaces [55].

#### 4.2.1. Description of reinforcement learning algorithms used.

The performance of each RL algorithm was evaluated against the PPO baseline and fixed-action strategies employed by other teams in the Autonomous Greenhouse Challenge. The virtual environment was enhanced to incorporate stochastic weather data generation, challenging the algorithms to adapt and generalize under dynamic and unpredictable conditions. A brief overview of the RL algorithms is provided below:

Proximal Policy Optimization (PPO): PPO leverages a policy-gradient approach with a clipped surrogate objective to ensure stable training. By limiting policy updates, it avoids large deviations that may destabilize learning [55].

Deep Deterministic Policy Gradient (DDPG): DDPG is an actor-critic algorithm de- signed for continuous action spaces. It employs deterministic policy updates alongside experience replay and target networks for stable training [56].

Soft Actor-Critic (SAC): SAC is an off-policy algorithm that optimizes a trade-off between expected reward and entropy, encouraging exploration. Its design makes it particularly effective in environments with stochastic dynamics [57].

Twin Delayed Deep Deterministic Policy Gradient (TD3): TD3 extends DDPG by addressing overestimation bias through twin Q-networks and improving stability with policy delay and target smoothing techniques [58].

#### 4.2.2. Training configuration and hyperparameters.

Each RL algorithm was configured with tailored hyperparameters to ensure robust performance and effective learning. Table 10 summarizes the key hyperparameters used for training, which include custom network architectures, entropy tuning, exploration noise, and soft target updates. These configurations facilitated consistent evaluation across all algorithms.

**Table 10:** Training Hyperparameters for RL Algorithms.

Parameter	TD3	SAC	DDPG	PPO
**Policy architecture**	Multi-Input	Multi-Input	Multi-Input	Multi-Input
**Network architecture**	[256, 256, 128]	[256, 256, 128]	[256, 256, 128]	[256, 256, 128]
**Learning rate**	1e-4	1e-4	1e-3	1e-5
**Discount factor**	0.99	0.99	0.99	0.99
**Batch size**	256	16	8	64
**Buffer size**	1,000	10,000	1,000	N/A
**Entropy coefficient**	N/A	Auto	N/A	0.05
**Train frequency**	1 episode	1 episode	1 episode	2048 steps
**Gradient steps**	100	23	1	20
**Tau**	0.005	0.005	0.002	N/A

To ensure a fair and reproducible comparison, all reinforcement learning algorithms (PPO, DDPG, SAC, and TD3) were trained under identical environments, observation spaces, episode horizons, and evaluation protocols, and were allocated the same computational budget in terms of total interaction steps and training duration. Hyperparameter tuning strategies and values are reported in Table 10.

Stochastic weather conditions were intentionally incorporated into the environment and regenerated at the beginning of each episode, exposing all agents to diverse environmental realizations during training and evaluation. This design enhances robustness and prevents overfitting to a single weather trajectory, while maintaining a consistent and fair comparison since all algorithms experience the same stochastic generation mechanism.

For each algorithm, extensive training and hyperparameter tuning were conducted, and the reported results correspond to a representative finalized configuration. The hyperparameters were selected to balance computational efficiency and learning performance. For instance, PPO’s small learning rate ensures training stability, while SAC’s automatic entropy tuning dynamically balances exploration and exploitation. Similarly, TD3 mitigates overestimation bias with twin Q-networks and policy delay, while DDPG enhances exploration through Ornstein-Uhlenbeck noise.

#### 4.2.3. Training process and monitoring.

The training process spanned 120,000 steps and involved iterative interactions with the environment. At each timestep, the RL agent observed greenhouse conditions, selected control actions based on its policy, executed the actions in the environment, and recorded the resulting states and rewards. Periodic policy and value updates were performed using experience replay buffers. To monitor progress, the following strategies were employed:

1. Evaluation Callback: Agents were evaluated every 10,000 steps, and the best- performing models were saved for reference.
2. Checkpoint Callback: Model checkpoints were saved every 10,000 steps, enabling recovery if necessary.
3. Visualization Tools: Training progress was visualized using TensorBoard, which logged metrics such as cumulative rewards, loss values, and episodic rewards.

#### 4.2.4. Performance evaluation.

After training, the RL algorithms were assessed based on their ability to optimize green- house control. Performance metrics included cumulative rewards, crop growth parameters, and resource consumption levels. The results were benchmarked against the PPO baseline and fixed-action strategies from the Autonomous Greenhouse Challenge. The analysis highlighted the effectiveness of trained RL agents in maximizing crop yields while maintaining resource efficiency under both static and dynamic environmental conditions. In summary the methodology integrates advanced ML and RL techniques for autonomous greenhouse control. Key innovations include a custom virtual environment simulating dynamic greenhouse conditions, feature selection to enhance model efficiency and RL agent performance, and a robust RL framework capable of optimizing greenhouse control strategies under varying environmental conditions.

## 5. Results

This section presents experimental results evaluating the RL-based framework designed to optimize greenhouse control strategies. The results are organized as follows: (1) performance evaluation of the PPO-baseline RL agent in different greenhouse environments with and without feature selection, and with/without random weather generation, (2) comparison of different RL algorithms during training, (3) comparative analysis of the best performed RL-based agent (TD3) against fixed-action strategies, and (4) evaluation of crop yield and resource consumption metrics. The findings are discussed in the context of their implications for sustainable agriculture.

### 5.1. Performance of PPO baseline RL agent

The PPO-RL agent’s performance was assessed over multiple training episodes, simulating complete growing cycles for cherry tomato crops. Key performance indicators included cumulative rewards, training results. The performance during training for the PPO-RL agent on both environments: (1) The full-features environment and the environment with feature selection, (2) environment with/without random weather generations were recorded.

#### 5.1.1. Evaluation with and without feature selection.

**Fig 23:**
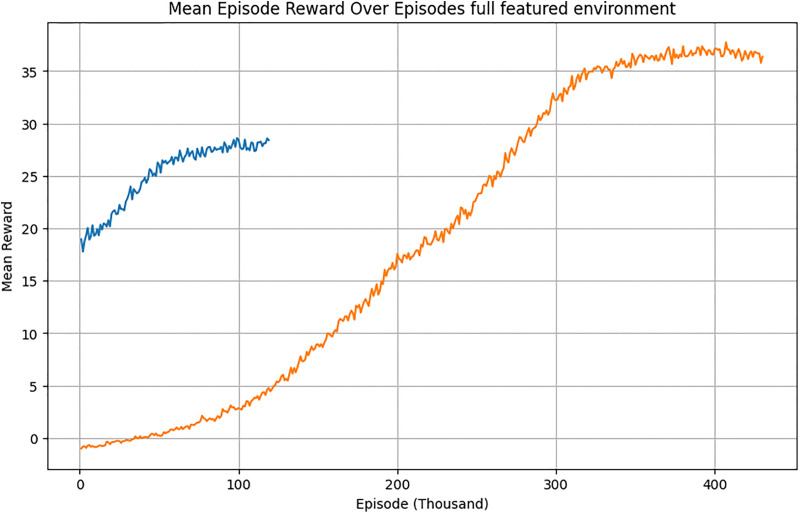
Cumulative rewards over episodes of full featured environment compared with environment with feature selection.

The PPO-RL agent underwent extensive training in environments with and without feature selection. The cumulative rewards shown in Fig 23 demonstrate the agent’s ability to learn and adapt to greenhouse conditions. Training in the feature-selected environment achieved optimal strategies in fewer episodes compared to the full-featured environment, reducing the total training time and complexity.

The feature-selected environment reduced training time and improved efficiency by focusing on critical features. The RL agent achieved optimal strategies more quickly, confirming the benefits of reduced complexity.

#### 5.1.2. Evaluation under stochastic weather.

**Fig 24:**
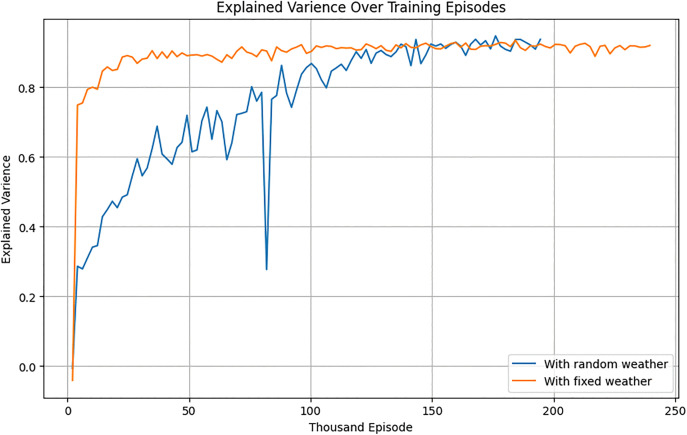
Explained variance of the RL agent over the training episodes for stochastic and deterministic environment.

**Fig 25:**
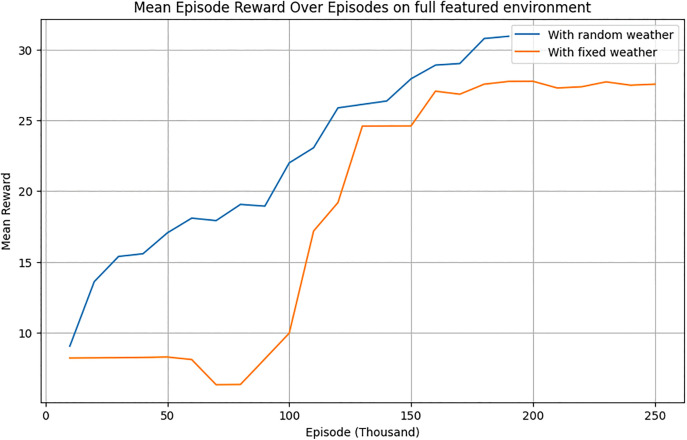
Evaluation mean-episode reward of RL-Agent for stochastic and deterministic environment.

**Fig 26:**
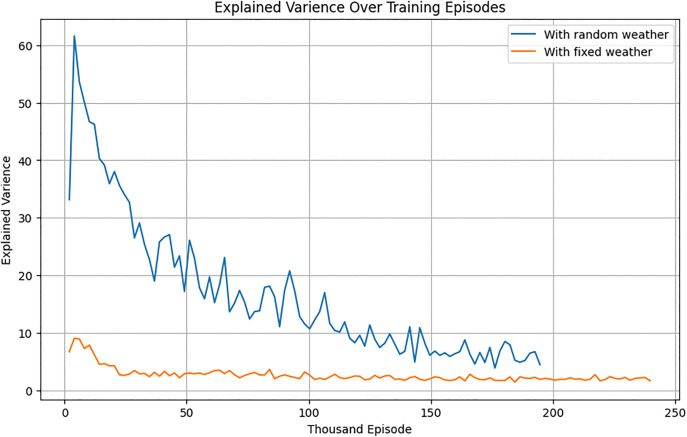
Training value loss of RL agent over the training episodes for stochastic and deterministic environment.

To evaluate robustness, the RL agent was tested under randomly generated weather conditions. Initially trained on historical weather data, the agent exhibited reduced performance under dynamic conditions. Retraining in the stochastic environment significantly improved its adaptability (Figs 24–26). After training, the RL agent was tested in a random weather generation environment to assess its robustness under dynamic conditions.

**Fig 27:**
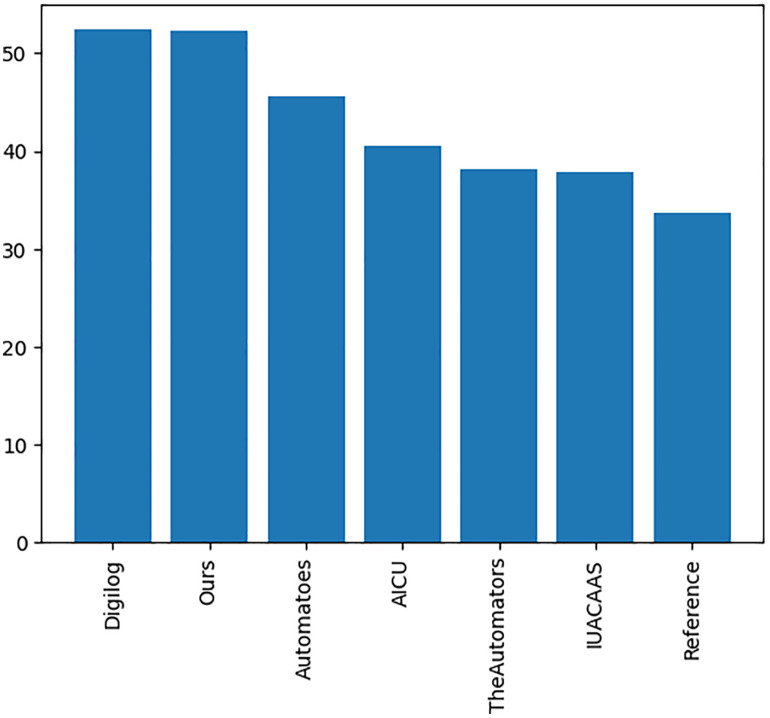
Accumulated rewards of PPO agent’s relative to the team strategies on historical weather Environment.

**Fig 28:**
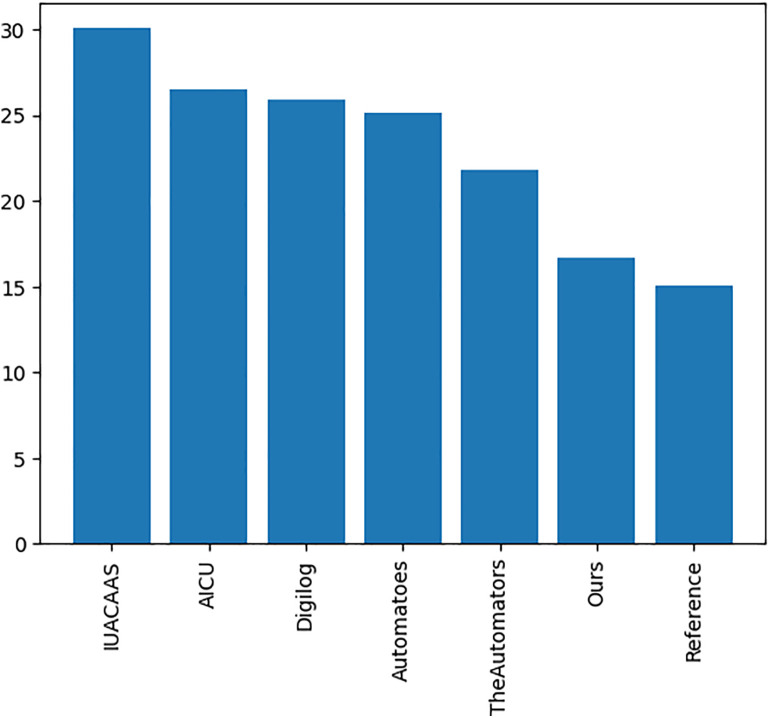
Accumulated rewards of PPO and team strategies under random weather conditions.

Fig 27 indicated that while the PPO agent outperformed team strategies under historical weather data, its performance declined under random weather conditions (Fig 28).

**Fig 29:**
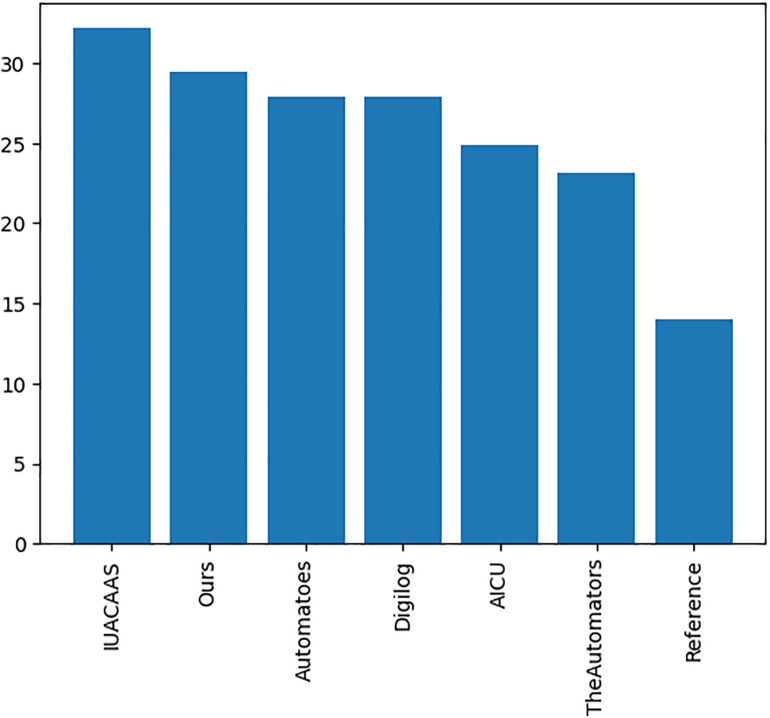
Improved rewards of the PPO agent after re-training in the random weather generation environment relative to the team strategies.

Re-training the PPO agent in the random weather environment significantly improved performance, as demonstrated in Fig 29, underscoring its ability to adapt to stochastic inputs.

### 5.2. Performance comparison between RL algorithms

This section compares the performance of four reinforcement learning (RL) algorithms (PPO, DDPG, SAC, and TD3) on the virtual greenhouse environment. The comparison focuses on their ability to maximize rewards, adapt to stochastic weather conditions, and optimize resource efficiency.

**Fig 30:**
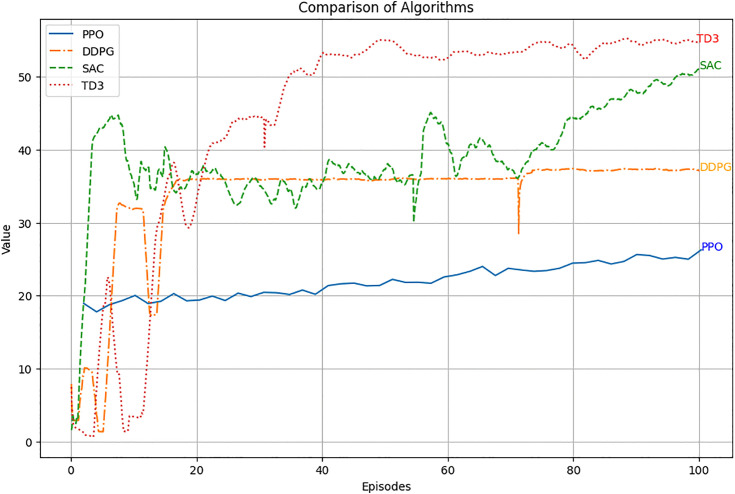
Rollout mean rewards over episodes for PPO, DDPG, SAC, and TD3.

**Fig 31:**
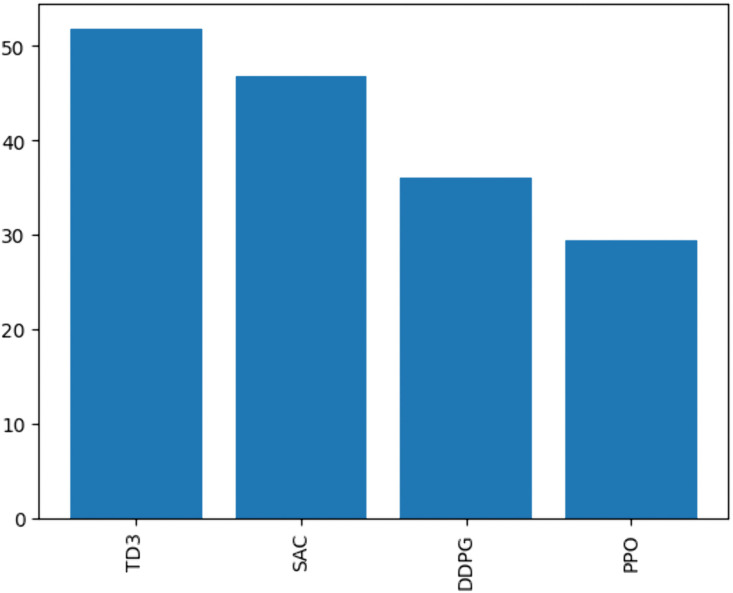
Average rewards achieved by each RL Algorithm.

The performance of PPO, DDPG, SAC, and TD3 algorithms was compared in the green- house environment. Fig 30 illustrates the rollout mean rewards over training episodes. TD3 achieved the highest rewards and stable convergence, outperforming SAC, DDPG, and PPO. The average rewards achieved by each algorithm are summarized in Fig 31, with TD3 leading at 51.8, followed by SAC (46.7), DDPG (36.0), and PPO (29.4).

The superior performance of TD3 in our experiments can be attributed to its algorithmic enhancements over baseline actor-critic methods. By employing twin Q-networks, TD3 mitigates overestimation bias common in continuous control, ensuring more reliable value estimates. Policy delay prevents premature updates that could destabilize training, while target policy smoothing reduces the impact of noise on policy updates. These features align well with the greenhouse control problem, where multi-variable coupling and actuator limits demand stable, incremental policy adjustments. Compared to SAC, TD3’s deterministic policy outputs allow for finer-grained control, and its exploration strategy better balances exploitation of known optimal setpoints with exploration under stochastic weather conditions.

### 5.3. Comparison analysis of best agent (TD3) with teams fixed strategies

The TD3 agent was benchmarked against fixed-action strategies from competing teams, with comparisons focusing on cumulative rewards, crop yield, and resource consumption. The comparative evaluation follows the setup of the 2nd International Autonomous Greenhouse Challenge, in which participating teams applied diverse AI and data-driven control algorithms, while a reference team operated the greenhouse manually using expert-defined fixed setpoints. Specifically, the participating teams (The Automators, AICU, IUA.CAAS, Digilog, and Automatoes) employed autonomous AI-based algorithms for climate, irrigation, and crop management, while the reference compartment reflected traditional expert-driven greenhouse operation. All teams are serving as the baselines for evaluating the efficiency of data-driven policies. the proposed TD3 agent’s control performance compared against both static data-driven and expert-controlled strategies, demonstrating superior adaptability and sustainability performance. The TD3 agent achieved the most favorable trade-off among all tested algorithms, realizing a 24.05% reduction in irrigation consumption while maintaining yield performance and only modestly increasing heating and electricity use.

#### 5.3.1. Cumulative rewards.

**Fig 32:**
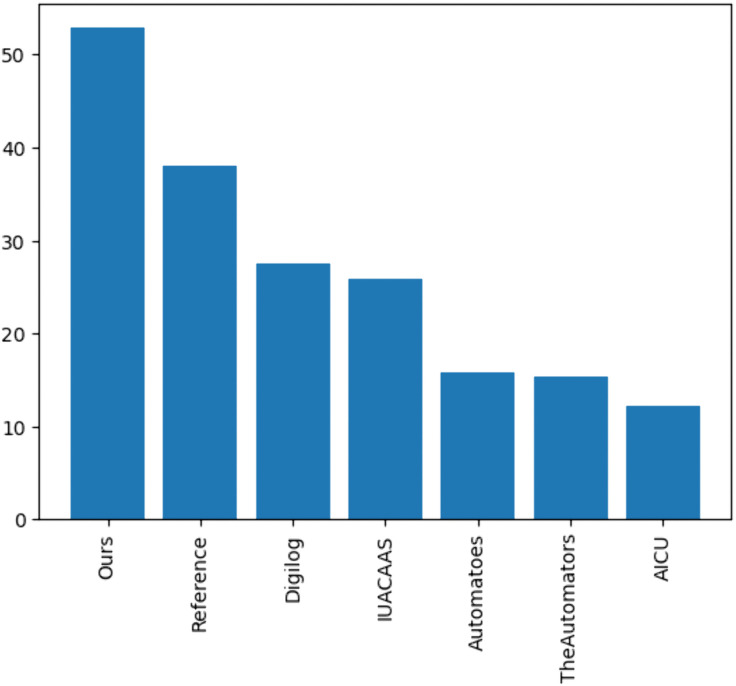
Cumulative rewards of TD3compared to team strategies.

The cumulative rewards for TD3 and teams strategies have been calculated, Fig 32 shows that the TD3 agent consistently achieved higher cumulative rewards compared to all team strategies, highlighting its adaptability and dynamic optimization capabilities.

#### 5.3.2. Crop yield parameters.

**Fig 33:**
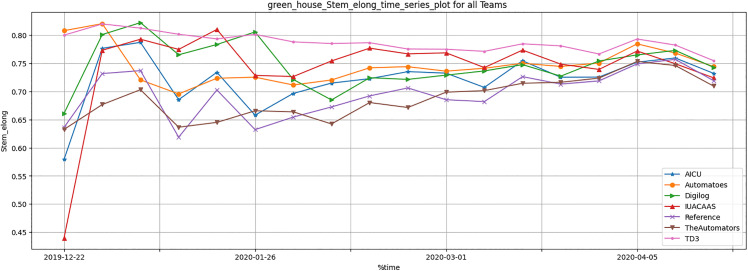
Weekly greenhouse stem elongation result during full episode for all teams.

**Fig 34:**
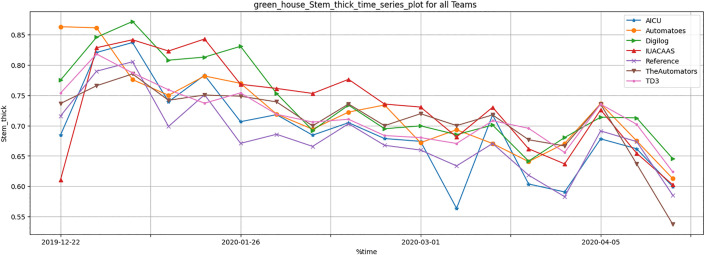
Weekly greenhouse normalized stem thickness results during full episode for all teams.

**Fig 35:**
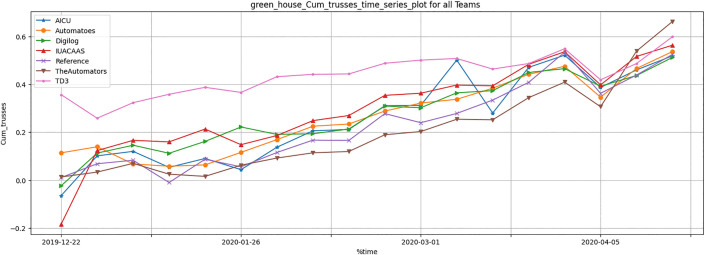
Weekly greenhouse normalized cumulative trusses results during full episode for all teams.

Table 11 summarizes crop yield metrics, including stem elongation, thickness, and cumu- lative trusses. The TD3 agent outperformed traditional strategies, achieving a significant improvement in cumulative trusses. The TD3 agent demonstrated significant improvements in crop yield parameters, particularly cumulative trusses, as illustrated in Figs 33–35. These improvements highlight the TD3 agent’s ability to optimize crop growth while balancing resource allocation.

**Table 11:** Average crop yield parameters across all teams for full episode.

Team	Stem elongation	Stem thickness	Cumulative trusses
**AICU**	0.73	0.70	0.26
**Automatoes**	0.75	0.73	0.27
**Digilog**	0.76	0.75	0.28
**IUACAAS**	0.75	0.74	0.30
**Reference**	0.71	0.69	0.24
**TheAutomators**	0.69	0.72	0.21
**Ours (RL Agent)**	**0.79**	**0.73**	**0.42**

#### 5.3.3. Resource consumption metrics.

**Fig 36:**
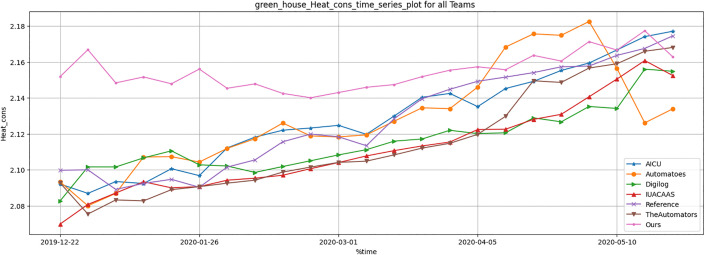
Greenhouse heat consumption during full episode for all teams.

**Fig 37:**
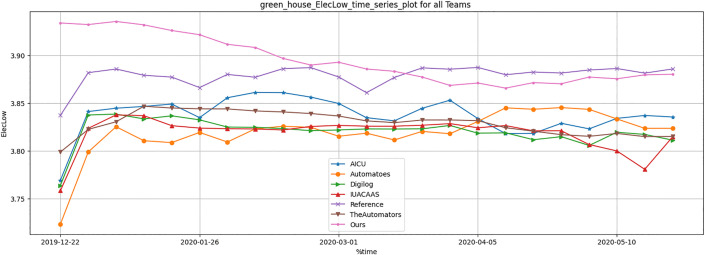
Greenhouse electricity low result during full episode for all teams.

**Fig 38:**
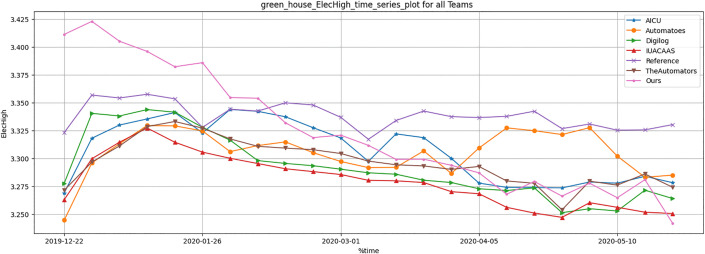
Greenhouse electricity high result during full episode for all teams.

**Fig 39:**
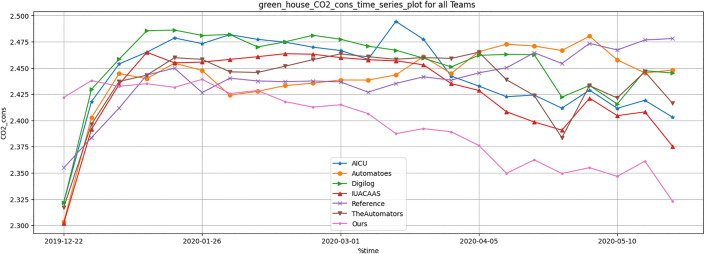
Greenhouse CO_2_ consumption during full episode for all teams.

**Fig 40:**
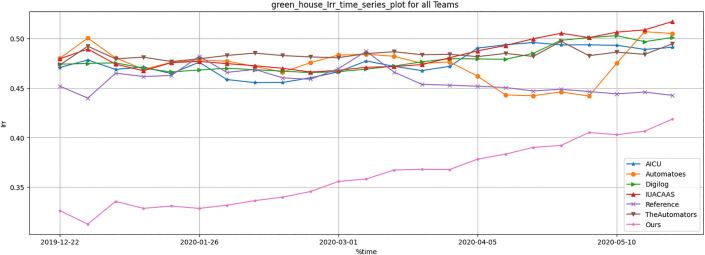
Greenhouse Irrigation result during full episode for all teams.

Resource usage metrics, including CO_2_, heat, electricity and irrigation of TD3 agent were compared across all strategies. Table 12 and Figs 36–40 show that TD3 agent maintained competitive resource efficiency while achieving higher crop yields. The adaptive nature of the TD3 agent allowed it to dynamically optimize resource use based on greenhouse conditions.

**Table 12:** Cumulative sum of resource usage across all teams.

Team	CO₂	Elec high	Elec low	Heat	Irrigation
**AICU**	56.20	76.05	88.26	48.95	10.96
**Automatoes**	56.15	76.02	87.84	48.95	10.90
**Digilog**	56.45	75.70	87.87	48.66	11.01
**IUACAAS**	55.84	75.44	87.83	48.56	11.13
**Reference**	56.11	76.79	89.22	48.93	10.52
**TheAutomators**	56.04	75.82	88.07	48.63	11.13
**Our RL Agent**	**55.09**	**76.45**	**89.58**	**49.56**	**8.31**

The RL-based approach demonstrated superior adaptability and efficiency in greenhouse control, achieving:

- Improved crop yields, particularly in cumulative trusses, compared to fixed-action strategies.
- Competitive resource efficiency across metrics with the TD3 agent achieving optimal resource utilization, particularly a 24.05% reduction in irrigation.
- Enhanced robustness under stochastic weather conditions after re-training.

The experimental results indicate that the Twin Delayed Deep Deterministic Policy Gradient (TD3) agent consistently achieved a balanced performance in resource utilization optimization compared to other benchmark strategies, as evaluated within the simulated greenhouse environment. The agent’s adaptive policy enabled dynamic decision-making, which resulted in satisfactory crop outcomes while effectively managing resource consumption.

Table 13 presents the calculated deviation ratios of the TD3 agent’s resource consumption relative to the average values reported by other participating teams. The deviation ratio for each resource was computed using the following formula:

**Table 13:** The resource consumption deviation ratios for TD3 agent relative to the average of other teams.

Parameter	TD3 agent Value	Average of other teams	Deviation ratio (%)
**CO₂ consumption**	55.09	56.13	–1.86
**Elec high**	76.45	75.97	+0.63
**Elec low**	89.58	88.18	+1.59
**Heat consumption**	49.56	48.78	+1.60
**Irrigation**	8.31	10.94	–24.05


$$Deviation\;Ratio\left(\%\right)=\frac{TD3Agent\;Value-Average\;of\;Other\;Teams}{Average\;of\;Other\;Teams}\times 100$$ (6)

The deviation ratios underscore the TD3 agent’s strategic approach to balancing resource efficiency with crop performance across key metrics. A deviation of −1.86% in CO2 consumption suggests a slightly more conservative dosing strategy, likely maintaining photosynthetic efficiency while reducing CO2 usage. The 0.63% increase in high electricity consumption may be attributed to targeted lighting during critical growth phases, while the 1.59% increase in low electricity usage reflects optimized energy management during off-peak periods. Additionally, the 1.6% rise in heat consumption indicates a focus on maintaining stable temperatures to support consistent crop yield quality. Most notably, the −24.05% deviation in irrigation highlights the agent’s ability to implement advanced water conservation measures without compromising crop productivity.

#### 5.3.4. Reward sensitivity analysis.

**Fig 41:**
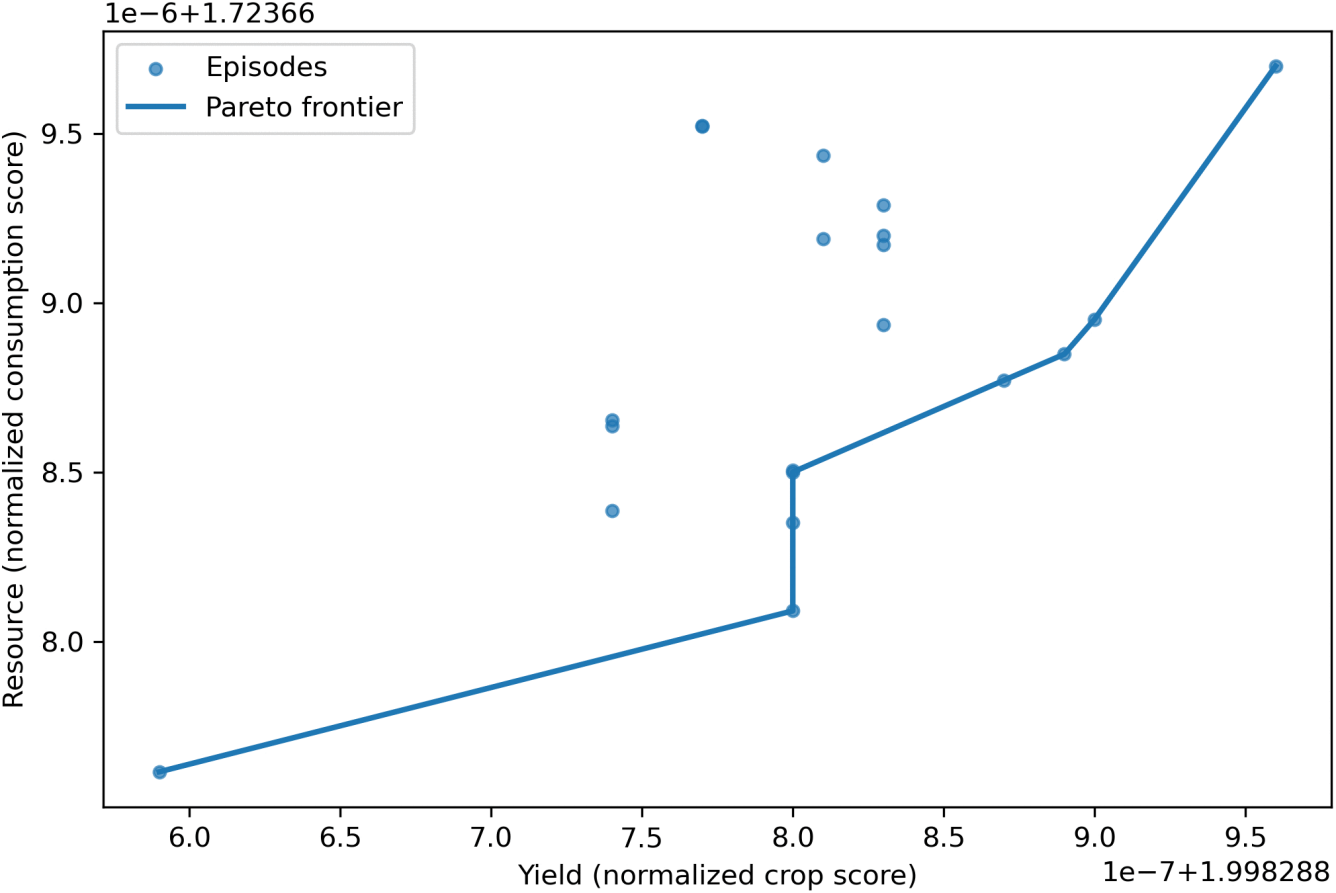
Pareto frontier of yield versus resource consumption under different reward weightings.

**Fig 42:**
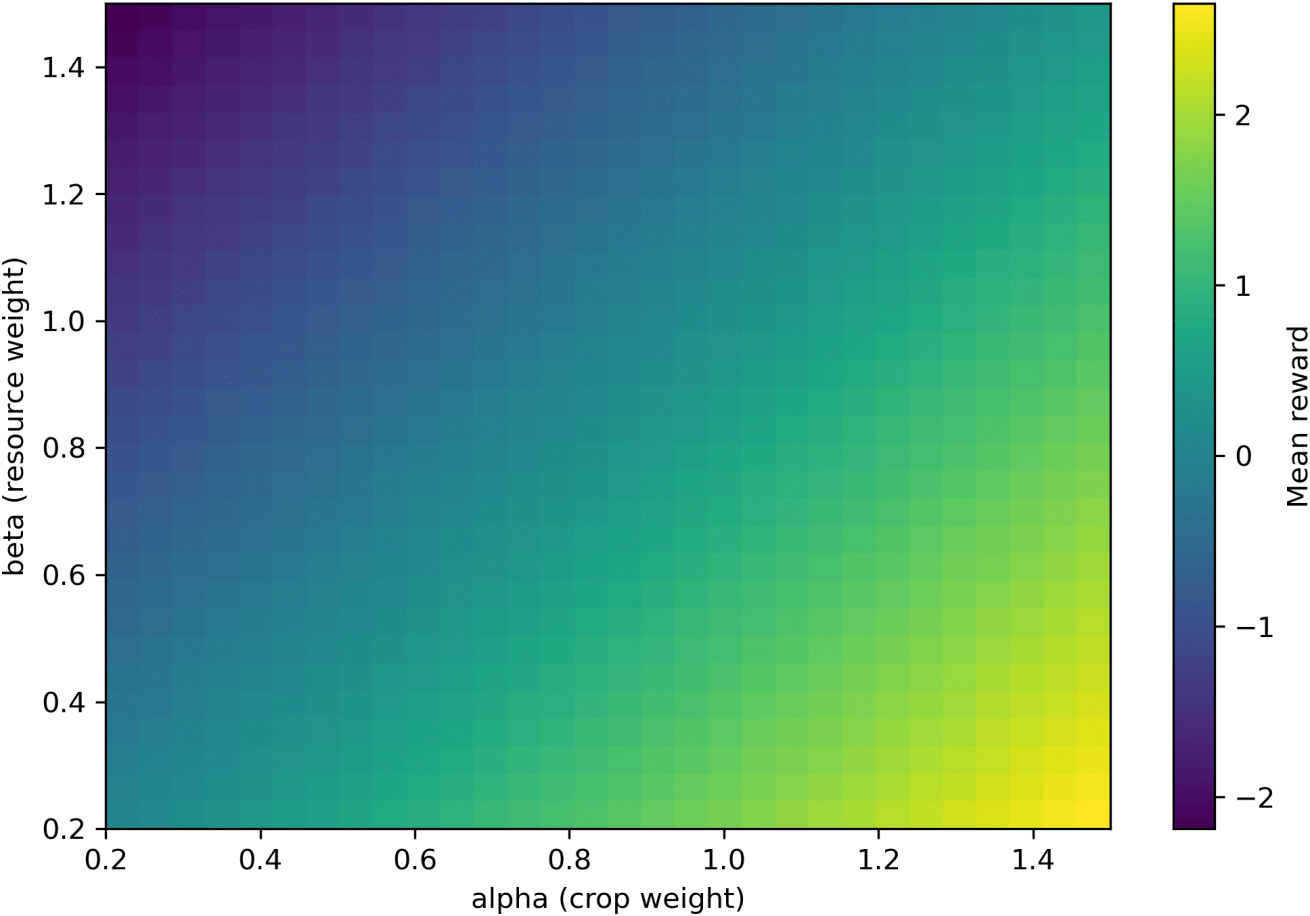
Sensitivity heatmap of average reward as a function ofα (yield weight) and β (resource weight).

To evaluate the robustness of the framework, we performed a structured sweep of the reward weights α (crop yield) and β (resource consumption). The Pareto frontier (Fig 41) highlights the trade-off between yield maximization and resource efficiency, with non-dominated solutions spanning multiple operating points. The corresponding heatmap (Fig 42) confirms that increasing α systematically favors yield-oriented policies, while higher β values promote resource-efficient behaviors.

The Pareto points are clustered within a narrow band, which is expected given the normalization of crop growth and resource caps. Within this compressed scale, the agent’s solutions delineated a consistent frontier, indicating that incremental yield gains necessarily required proportional increases in resource use. Complementary sensitivity analysis further confirmed this observation, as varying (α, β) shifted the mean reward surface smoothly, showing robustness to different preference settings.

These results indicate that the observed performance gains are not driven by a narrowly tuned choice of α and β but remain stable across a range of yield-resource trade-off configurations, demonstrating robustness with respect to the primary reward weights.

In summary, the TD3 agent achieved a favorable trade-off between resource usage and yield optimization. Minor increases in specific parameters (e.g., heating and electricity) are justified by their role in enhancing crop growth conditions. The significant reduction in irrigation consumption stands out as a key indicator of the agent’s potential in advancing sustainable greenhouse management. These findings affirm the applicability of reinforcement learning in optimizing resource use while supporting productive and sustainable agricultural practices.

## 6. Discussion

This study evaluates the application of a reinforcement learning (RL) based framework for optimizing greenhouse control strategies, addressing the complexities of managing dynamic environments. The RL agent demonstrated notable improvements in crop growth and resource efficiency, highlighting its potential to advance sustainable agricultural practices. Among the RL algorithms tested, the TD3 agent consistently outperformed others, achieving higher cumulative rewards, better crop yield metrics, and balanced resource usage. The findings align with existing literature on AI-driven agricultural management while extending its scope to include a unified multi-objective RL framework.

The RL agent displayed several strengths that underscore its effectiveness in greenhouse management:

- Crop Growth Management: The TD3 agent achieved significant improvements in crop metrics, such as stem elongation, stem thickness, and cumulative trusses. The TD3 agent consistently outperformed other strategies in cumulative trusses, highlighting its ability to optimize crop yield effectively.
- Resource Efficiency: The TD3 RL agent exhibited balanced resource utilization, achieving reductions in CO_2_ and irrigation consumption while maintaining competitive electricity and heat usage. These results emphasize the agent’s capability to optimize resource allocation for sustainability.
- Reward Maximization: The TD3 agent attained the highest cumulative rewards among all algorithms, reflecting its ability to optimize trade-offs between crop growth and resource efficiency dynamically.

### 5.4. Implications for greenhouse management

The study highlights the potential of RL-based systems to revolutionize greenhouse management by optimizing crop yields and resource use. These AI-driven approaches address the dual challenges of global food demand and climate change by enhancing sustainability in agricultural practices. The integration of RL into autonomous greenhouses represents a significant step toward efficient, scalable, and sustainable farming systems.

### 5.5. Training performance of PPO baseline

The Proximal Policy Optimization (PPO) algorithm served as the baseline for evaluating RL performance. The agent’s training results revealed the following:

- Rollout Mean Rewards: As shown in Fig 30, the PPO agent displayed a steady upward trend in cumulative rewards across episodes, indicating effective learning and policy convergence. However, its performance lagged behind that of the TD3 agent due to its conservative policy updates.
- Explained Variance: Stabilization near 0.8 (Fig 24) suggests that the PPO agent accurately predicted outcomes based on its policy, ensuring effective learning and decision-making.
- Value Loss: A steep initial reduction in value loss, followed by gradual stabilization (Fig 26), demonstrated the PPO agent’s ability to minimize errors over time.

While the PPO agent performed competitively, robust algorithms were necessary to train and test to surpass leading teams such as “IUACAAS” (Fig 29).

### 6.3. Cumulative rewards and strategy comparison

1. Impact of Random Weather Generation: Testing the PPO agent and fixed-action strategies under randomized weather conditions (Fig 28) revealed a decline in performance across all strategies. This finding highlights the limited adaptability of fixed-action policies and underscores the importance of refining RL-based approaches for stochastic environments.
2. Re-Training Under Random Weather: Re-training the RL agent under these randomized weather conditions significantly enhanced its robustness, allowing it to outperform fixed-action strategies in varying environments (Fi 29). This improvement demonstrates the RL agent’s capacity to generalize under dynamic conditions, a critical requirement for real-world greenhouse applications.
3. Fixed-Action Strategy Robustness: Fixed-action strategies, while effective under historical weather data, showed significant performance drops in randomized environments. This limitation validates the advantage of adaptive AI techniques, such as reinforcement learning, in managing complex systems.

### 6.4. Performance comparison between RL algorithms

The comparative analysis of PPO, DDPG, SAC, and TD3 revealed significant insights into the strengths and limitations of each algorithm.

The TD3 agent demonstrated the best performance, achieving the highest and most stable cumulative rewards (Figs 30,31). Its twin Q-networks, policy delay, and target smoothing mechanisms enhanced stability and optimization under complex greenhouse constraints. SAC performed well, with rewards close to TD3. Its entropy-based exploration enabled effective adaptation to stochastic conditions, though its policy updates were less efficient in some scenarios.

DDPG struggled with exploration in high-dimensional action spaces, resulting in lower rewards and less stable learning compared to TD3 and SAC. While PPO exhibited stable learning, its conservative policy updates limited its reward improvement, making it less effective than TD3, SAC and DDPG.

These findings reinforce the suitability of TD3 for complex greenhouse systems where nonlinear interactions and continuous action spaces dominate. Unlike PPO, which converges conservatively, or DDPG, which is prone to instability, TD3 achieves a balance between stability and adaptability, making it more resilient under dynamic climate conditions. This aligns with recent control engineering research emphasizing the reduction of function approximation error and the stabilization of policy updates in safety-critical environments.

In direct comparison, PPO provides stable but overly cautious updates; DDPG, though designed for continuous spaces, suffers from noisy and unstable exploration; and SAC improves adaptability through entropy maximization but often converges more slowly. By contrast, TD3 integrates deterministic policy outputs with stability-enhancing mechanisms such as twin critics, policy delay, and target smoothing. These properties produce reliable value estimates, smoother control trajectories, and enhanced robustness under both regular and stochastic climate inputs. Importantly, TD3’s smooth deterministic actions also reduce actuator wear and prevent abrupt microclimate changes, which are critical for crop health. This comparative analysis underscores why TD3 not only achieved superior numerical performance in our results but also aligns most closely with the operational requirements of real-world autonomous greenhouse management.

### 6.5. TD3 comparison with fixed-action strategies

The TD3 agent was compared against fixed-action strategies used by teams in the Autonomous Greenhouse Challenge, with the following observations:

Crop Yield Parameters: The TD3 agent consistently outperformed all teams in cumulative trusses (Fig 35), reflecting its ability to dynamically adapt resource usage to optimize plant growth. Teams such as “The Automators” and “Reference” fell behind due to suboptimal resource distribution strategies.

Resource Usage Optimization: The TD3 agent demonstrated efficient resource utilization, with notable reductions in irrigation (24.05% lower than the average of other teams) and CO_2_ consumption (1.86% lower than the average). These results underscore the agent’s capacity for sustainable water and energy management (Figs 36–40, Table 12).

Although our experiments did not explicitly simulate continuous extreme events such as prolonged rain or sandstorms, the stochastic weather generator already introduced large and irregular fluctuations in temperature, radiation, and humidity. These fluctuations represent a proxy for stress-testing, as they deviate substantially from normal seasonal variations and require the RL agent to adapt dynamically. The results (Figs 24–29) show that the retrained TD3 agent maintained stable performance despite these abrupt changes, underscoring its inherent climate resilience.

In particular, the stochastic weather generator used in this study incorporates high-variance patterns such as multi-day heatwaves, cold spells, and prolonged low radiation periods, which emulate real extreme climate events encountered in greenhouse operations. This ensures that the agent’s robustness is evaluated under both typical and stress-inducing environmental scenarios.

In practical terms, climate extremes can be considered as extended versions of the fluctuations already modeled in our randomized scenarios. The ability of the RL agent to generalize under such irregular dynamics provides confidence in its robustness. Future work will extend this by incorporating disaster-oriented scenarios (e.g., multi-day rain, heat waves, or sandstorms) following the approaches in recent resilience-focused greenhouse studies, thereby quantifying the agent’s performance under rare but high-impact events.

Prediction errors from the MLP-based climate model and the LSTM-based crop and resource models propagate into the reinforcement learning loop as structured noise rather than deterministic bias. To mitigate potential error amplification, the RL agent was trained under stochastic weather perturbations and evaluated using reward sensitivity and Pareto-front robustness analyses. This design ensures that learned policies remain stable despite moderate prediction inaccuracies, as evidenced by consistent convergence behavior and smooth trade-offs across varying reward weight configurations.

### 6.6. Trade-off and sensitivity robustness

The sensitivity of the proposed framework to reward weights was systematically investigated to assess how different values of the coefficients α (crop yield weight) and β (resource consumption weight) influence the learned trade-off between productivity and sustainability. Unlike exploratory tuning, we performed a structured sweep of α and β across a defined grid and evaluated performance using logged evaluation episodes.

The Pareto frontier analysis (Fig 41) clearly illustrates the inherent trade-off: higher yields are only attainable at the cost of increased resource consumption, and vice versa. This confirms that the framework naturally spans multiple operational regimes that stakeholders can select based on strategic priorities.

To further quantify sensitivity, a heatmap of mean reward across (α, β) configurations was generated (Fig 42). The results show that increasing α systematically shifts the policy toward yield-maximizing behaviors, whereas higher β values promote resource efficiency. The transition is smooth and continuous, underscoring the robustness of the reward design. Importantly, the reward landscape does not collapse into degenerate solutions, suggesting that the formulation generalizes well across a spectrum of stakeholder preferences.

The limited spread of points on the Pareto plots should not be interpreted as a lack of variability, but rather as a consequence of normalization and intrinsic greenhouse constraints. This concentration highlights the stability of the RL agent, which consistently identifies balanced strategies. The complementary heatmap further supports this robustness, showing that systematic adjustments to α and β produce interpretable shifts in the yield–resource balance rather than unstable or erratic responses.

Together, Figs 41 and 42 demonstrate that the proposed framework provides a structured and tunable trade-off frontier that adapts to diverse sustainability objectives. These findings directly address the reviewer’s concern, confirming that the reward function offers a flexible and universal mechanism to balance productivity with resource efficiency in practical greenhouse management.

### 6.7. Algorithmic interpretation and deployment implications

The TD3 agent’s twin-critic structure mitigates overestimation of aggressive CO₂ dosing, avoiding waste. Policy-delay prevents abrupt irrigation or heating oscillations that could stress plants, while target-policy smoothing stabilizes exploration under stochastic weather. Table 14 summarizes behavioral differences among TD3, DDPG, and SAC.

**Table 14:** Behavioral comparison of RL algorithms under stochastic greenhouse control.

Algorithm	Key architectural feature	Observed learning behavior	Resource-use Pattern	Policy stability
**DDPG**	Single critic, no delay	Fast initial learning but frequent value overestimation	High variance in CO₂ and irrigation control	Occasional oscillations
**SAC**	Entropy-regularized stochastic policy	Stable but slower convergence; higher exploration noise	Moderate efficiency; delayed stabilization	Medium stability
**TD3 (proposed)**	Twin critics, policy delay, target smoothing	Smooth convergence with low variance; consistent long-term policy	~24% reduction in irrigation with marginal energy increase	High stability; robust to weather noise

To further illustrate these behaviors, a comparative evaluation of TD3, DDPG, and SAC agents was conducted using the same stochastic-weather greenhouse environment. TD3 exhibited markedly smoother policy convergence and fewer control oscillations in both irrigation and heating setpoints. In contrast, DDPG occasionally overestimated aggressive CO₂ dosing, leading to transient spikes in resource use, while SAC showed slower convergence and higher variance during exploration. These observations align with the theoretical strengths of TD3’s architecture, its twin-critic stability and delayed policy updates, which enable consistent decision-making under biological and environmental uncertainty.

### 6.8. Alignment with existing literature

This research aligns with and expands upon existing studies in AI-driven agricultural management. Unlike prior works focusing on single objectives such as irrigation or energy optimization, this study integrates multi-objective RL to simultaneously optimize crop growth and resource efficiency.

By incorporating dynamic weather generation and multi-variable greenhouse control, the study addresses limitations in traditional approaches that rely on static environments or predefined setpoints. The TD3 agent’s superior performance builds on foundational works by demonstrating adaptability to stochastic conditions and robustness in optimizing crop yield and resource use.

### 6.9. Cross-disciplinary universality

The principles underlying our RL-based resource optimization extend beyond agriculture and are applicable to other dynamic, resource-constrained systems. Similar challenges exist in energy systems, manufacturing, and process industries, where sustainability optimization requires balancing efficiency, performance, and resource recovery. For example, the reversible loss recovery model for battery systems presented in [59] provides a theoretical basis for managing the balance between consumption and recovery in real time. Drawing parallels to greenhouse management, incorporating intermittent recovery phases for heating and CO₂ supply equipment could further enhance system sustainability. Such cross-disciplinary perspectives underscore the universality of our framework and open pathways for integrating methodologies from other domains to strengthen climate-resilient agricultural practices.

### 6.10. Practical deployment potential

Practical deployment of the proposed framework can be achieved by integrating the RL agent with existing IoT-based greenhouse management systems. Real-time sensor data can be streamed to a cloud-hosted RL service, which computes optimal setpoints and communicates them to local actuator controllers.

The full deployment loop operates as follows: (1) sensors measure temperature, humidity, and CO₂ concentration; (2) a local edge device runs the MLP model for immediate climate state estimation; (3) state data and short-term forecasts from the LSTM models are transmitted to the cloud-based TD3 agent for decision-making; (4) the agent computes optimal weekly setpoints for heating, ventilation, and irrigation; and (5) these setpoints are returned to the edge controller, which executes the actions through the actuator interface. The combined inference footprint of the MLP-LSTM-TD3 pipeline remains lightweight (<50 MB RAM and <0.1 s per forward pass on an NVIDIA Jetson Nano), making deployment feasible on low-power edge hardware or hybrid cloud–edge systems. In case of communication loss or model failure, the rule-based controller automatically re-engages to maintain safe operating limits and prevent crops or equipment damage.

Periodic retraining with updated data would ensure adaptability to seasonal changes and equipment aging. This deployment pathway supports both on-premises and remote decision support, making it viable for large-scale commercial adoption.

In real-world deployments, additional operational challenges must be considered. Sensor noise and measurement drift may introduce uncertainty into state estimation, which can be mitigated through sensor calibration routines, redundancy, and filtering techniques at the edge level. Actuator delays and response latencies, particularly in heating and ventilation systems, may affect control precision and should be accounted for through conservative action constraints and delayed-reward awareness during training. Model retraining frequency represents a trade-off between adaptability and operational stability; in practice, retraining can be scheduled periodically or triggered by performance degradation indicators. From a computational perspective, the lightweight inference footprint enables on-site deployment on embedded hardware, while more computationally intensive retraining processes can be performed offline or in the cloud.

While the proposed RL framework demonstrated robust performance under stochastic environmental conditions, long-term deployment must consider gradual equipment performance degradation. For example, heating system efficiency and CO₂ dosing accuracy may deteriorate over extended operation periods. Integrating predictive maintenance models, such as the dynamic operating life prediction framework described in (50) could enable the RL agent to adjust control strategies based on real-time equipment health indicators, ensuring sustained optimization.

### 6.11. Limitations

While the study demonstrates the effectiveness of RL-based frameworks for greenhouse management, certain limitations remain. The agent’s performance is influenced by the quality and diversity of training data. Limited training scenarios may reduce adaptability to unforeseen conditions. The RL agent was tested in a virtual simulation. Real-world implementation may face challenges related to data collection, sensor accuracy, and integration with existing infrastructure.

Furthermore, the experimental validation relies on a single crop (cherry tomato) and a specific high-tech greenhouse dataset, which may limit the direct generalizability of the learned control policies to other crops, greenhouse configurations, or climatic regions without additional retraining or adaptation.

Although stochastic environmental conditions are regenerated across episodes to enhance robustness and each algorithm underwent extensive training and hyperparameter tuning, the reported results summarize representative performance from a single finalized training configuration per algorithm. Future work could further strengthen statistical confidence by aggregating performance across multiple independent training runs.

In addition, while our study employed LSTM models for time series forecasting of crop parameters and resource consumption, recent advances suggest that Transformer-based architectures may offer superior capability in modeling long-term and irregular environmental fluctuations. As highlighted in [60], 3050, future work comparing LSTM and Transformer models could provide further improvements in adaptability, particularly under extreme or unpredictable climate conditions.

Future research could explore model-based reinforcement learning (MBRL) frameworks, leveraging predictive models to simulate interactions and guide RL training. This approach could enable scalable, data-efficient training while ensuring adaptability to dynamic real- world conditions.

This study highlights the potential of RL-based systems to modernize greenhouse management by achieving a balance between crop yield optimization and resource efficiency. The TD3 agent’s superior performance underscores the transformative role of AI in advancing sustainable agricultural practices. By addressing existing limitations and expanding its scope, this research paves the way for scalable and adaptive solutions in response to global challenges in food security and climate change.

## 7. Conclusions

This study highlights the transformative potential of reinforcement learning (RL) in advancing autonomous greenhouse management systems. By dynamically optimizing critical parameters for crop growth and resource consumption, the proposed RL-based framework addresses pressing challenges in modern agriculture, including food security and resource sustainability.

The RL agent demonstrated its ability to adapt to varying environmental conditions, balancing short-term goals of high productivity with long-term sustainability. The agent effectively optimized control strategies to maximize crop yields while ensuring efficient re- source utilization.

Among the tested RL algorithms, the Twin Delayed Deep Deterministic Policy Gradient (TD3) agent emerged as the most robust, consistently achieving the highest cumulative re- wards, superior crop yield parameters (stem elongation, thickness, and cumulative trusses), and optimized resource consumption metrics (heat, electricity, CO_2_, and irrigation). Its twin-critic structure, policy delay, and smoothing mechanisms enabled reliable control under complex and stochastic greenhouse conditions, outperforming PPO, DDPG, and SAC. These results confirm TD3’s suitability for managing the dynamic and nonlinear nature of greenhouse environments, where both stability and adaptability are critical.

The RL agent outperformed fixed-action strategies under historical weather conditions and demonstrated significantly higher adaptability when trained in randomized weather environments. The retrained agent achieved higher cumulative rewards, showcasing its ability to generalize and respond to stochastic changes, a critical advantage over static strategies.

The TD3-based greenhouse controller achieved an effective balance between maximizing crop yield and minimizing resource consumption, demonstrating clear improvements over fixed-action strategies. Sensitivity analyses verified the universality and stability of the reward design, confirming adaptability to different sustainability priorities. The framework’s modular design and cross-domain applicability position it as a scalable step toward intelligent, sustainable agriculture.

A promising extension of this work lies in the integration of Model-Based Reinforcement Learning (MBRL) approaches. By leveraging predictive environment models, MBRL could enable more efficient training and deployment of RL agents in real-world greenhouse systems. This approach ensures continuous improvement and adaptability, bridging the gap between simulation and practical applications.

In conclusion, the proposed RL framework showcases its ability to optimize greenhouse operations by dynamically adapting to environmental conditions. It sets the foundation for future advancements in smart agriculture, paving the way for scalable, sustainable, and efficient agricultural practices.

## 8. Recommendations

To further enhance the applicability, robustness, and scalability of RL-based frameworks in autonomous greenhouse management, the following recommendations are proposed:

- Practitioners should implement RL-based systems to optimize decision-making processes and resource usage. Training programs should be developed to equip operators with the necessary skills to leverage AI-driven systems effectively, enabling dynamic and adaptable control over greenhouse conditions.
- To maintain adaptability, RL agents must undergo regular retraining using updated data reflecting changing environmental conditions and crop-specific requirements. This iterative process ensures sustained high performance and addresses evolving agricultural demands.
- RL agents should be trained with stochastic elements, such as random weather generation, pest infestations, and resource constraints, to improve their robustness under real-world uncertainties. Incorporating these dynamic scenarios during training enhances the agent’s generalizability and reliability in practical applications.
- Future research should refine the virtual greenhouse environment by incorporating diverse historical greenhouse records and conducting real-world benchmarking experiments. Advanced validation techniques, such as cross-validation with diverse datasets and sensitivity analyses, can further improve the model’s accuracy and reliability.
- The integration of IoT sensors into greenhouse systems can provide real-time data on critical environmental parameters such as temperature, humidity, soil moisture, and CO2 levels. This data enables the RL agent to make informed decisions and adapt dynamically to changes, creating a real-time feedback loop for precise resource management.
- Expanding RL frameworks to include diverse crop species, regional conditions, and growth environments can validate their scalability and adaptability. This approach ensures applicability across various agricultural systems, promoting broader adoption of AI-driven practices.
- Interdisciplinary collaborations with agronomists, environmental scientists, and agricultural practitioners are essential for refining RL models. Experts can provide insights into factors such as pest management, nutrient cycles, and soil health, ensuring the RL framework addresses real-world complexities effectively.
- Future research should explore the integration of MBRL, which enables simultaneous training of the RL agent and the environment model. This approach allows the system to dynamically adapt to new data and scenarios, bridging the gap between virtual simulations and practical agricultural applications.
- Real-world implementation of RL frameworks is critical for validating their performance and refining their effectiveness. Controlled greenhouse experiments can provide valuable insights into the practical utility of these systems, ensuring their readiness for large-scale deployment. Additionally, the assumption of uniform soil properties simplifies real-world variability. Incorporating heterogeneous soil-sensor feedback will enable spatially adaptive irrigation and temperature control.
- RL-based systems should be leveraged to enhance sustainability in agricultural practices. By optimizing resource usage and improving crop yields, these systems contribute to global efforts to address food security and climate change challenges.

By addressing these future directions, the proposed RL framework can be refined and expanded, contributing to a sustainable transformation of agricultural practices in response to global food production challenges.

## Supporting information

S1 File Supporting information. (Supporting Information.ZIP)
